# Comparison and Analysis of Resistance Differences in *Alternaria alternata* from Fungicides with Three Different Mechanisms

**DOI:** 10.3390/jof11040305

**Published:** 2025-04-11

**Authors:** Qian Bai, Xinbo Ma, Mansoor Hayat, Yuxin Tang, Zhanbin Wang

**Affiliations:** 1 School of Forestry, Northeast Forestry University, Harbin 150040, China; 2 Northeast Asia Biodiversity Research Center, Northeast Forestry University, Harbin 150040, China

**Keywords:** *Alternaria alternata*, prochloraz, mancozeb, fludioxonil, physiological indicators, transcriptome, resistance genes, resistance pathways

## Abstract

The pathogen *Alternaria alternata* infects a variety of plants and crops, notably poplars, and results in large financial losses. Using twelve chemical fungicides for fungicide sensitivity tests (FSTs) on *A*. *alternata*, the result showed that prochloraz (PCZ), mancozeb (MZ), and fludioxonil (FLU) have potent inhibitory effects against the pathogen through different mechanisms. To investigate how the pathogen responded to fungicide-induced stress, transcriptome and physiological investigations were carried out after treatments with three fungicides at their corresponding 50% effective concentration (EC_50_) doses. The MZ treatment produced a distinct genetic response; FLU treatment produced the greatest number of differentially expressed genes (DEGs), followed by PCZ. DEGs from FLU treatment were mostly engaged in ribosome biosynthesis, those from MZ treatment in lipid and carbohydrate metabolism, and those from PCZ treatment in carbohydrate metabolism, according to Gene Ontology (GO) analysis. Kyoto Encyclopedia of Genes and Genomes (KEGG) pathway enrichment analysis revealed that FLU and PCZ treatments were associated with ribosome biogenesis, whereas MZ treatment was linked to the pyruvate metabolic pathway. Collinear trend analysis indicates that MZ exhibits a unique pattern, with FLU treatment causing the most significant overexpression of genes, followed by PCZ. The six categories of 88 elevated DEGs associated with fungal resistance include tyrosinase, ATP-binding cassette (ABC) transporters, major facilitator superfamily (MFS) transporters, antioxidant and cellular resilience genes, as well as genes involved in cell wall and membrane biosynthesis. Notably, the pathways involved in the synthesis of melanin and ergosterol exhibited the strongest response to FLU. The results of a correlation analysis between physiological indices and resistance-related genes indicated that melanin content, malondialdehyde (MDA) content, and tyrosinase activity were positively correlated with the majority of resistance-related DEGs, whereas soluble protein content, superoxide dismutase (SOD) activity, and catalase (CAT) activity were negatively correlated, which is consistent with the observed trends in the measured physiological indicators. Taken together, this study provides a theoretical basis for developing more effective fungicides and chemical control strategies against *A. alternata*.

## 1. Introduction

*Alternaria alternata* is responsible for infecting various plants and crops, including poplars, causing severe economic losses [1,2]. Developing effective strategies for controlling *A. alternata* has become a critical and widely discussed issue in current research. As global emphasis on sustainable resource utilization increases, the use of pollution-free biological control methods to manage plant diseases becomes more prevalent. However, the use of chemical fungicides is still the main method for controlling plant diseases at present, owing to the slow effectiveness of biological control methods and the incomplete development of biological control technology.

Many cost-effective chemical fungicides have been developed through studies on the cellular structures and major metabolic pathways of pathogens to control plant diseases effectively and economically. Most of these fungicides act on microorganisms by interfering with metabolic pathways such as nucleotide synthesis, thereby inhibiting DNA/RNA synthesis, further suppressing protein synthesis, and disrupting enzymes involved in the synthesis of cell walls or cell membranes [3]. Understanding how fungicides act on plant pathogens is crucial for effective disease control and efficient fungicide application. Recent transcriptomic studies on fungicide resistance in fungal pathogens have revealed key molecular mechanisms underlying resistance evolution, including gene expression changes, metabolic adaptations, and efflux transporter upregulation [4,5,6]. Investigating the application methods of fungicides with different modes of action can enhance fungicidal efficiency while avoiding the simultaneous use of fungicides with similar mechanisms. This strategy can help reduce economic costs and labor inputs, thereby improving the overall sustainability of disease management practices.

Although chemical fungicides can immediately suppress plant diseases, their prolonged usage has caused plant pathogens to progressively adapt and acquire multidrug resistance to survive in such unfavorable environments. Multidrug resistance (MDR) refers to the resistance of microorganisms to antimicrobial agents that are structurally unrelated and have different molecular targets [7]. The emergence of multidrug resistance diminishes the effectiveness of chemical control of plant diseases and increases control costs. Transcriptomic analyses of fungicide-resistant fungal strains have identified differentially expressed genes involved in efflux pump overexpression, detoxification pathways, and stress response mechanisms in pathogens such as *Botrytis cinerea*, *Zymoseptoria tritici*, and *Fusarium graminearum* [8,9,10,11,12,13]. Current research on fungal drug resistance mechanisms emphasizes the importance of resistance-mediated drug efflux transporters. Fungi can survive in the presence of poisonous substances because these transporters can prevent the buildup of harmful amounts of compounds inside their cells [14]. The resistance of major fungal pathogens to multiple chemical fungicides is often linked to the overexpression of efflux pumps, which actively transport toxic compounds out of cells [15]. Among these, ATP-binding cassette (ABC) transporters and major facilitator superfamily (MFS) transporters play key roles. ABC transporters utilize ATP hydrolysis to drive drug efflux, whereas MFS transporters function as secondary active transporters that mediate multidrug resistance (MDR) through proton gradients [16,17,18,19]. Efflux-mediated resistance has been widely reported in fungal pathogens, including *A. alternata* [20]. However, the specific regulatory mechanisms governing the expression and activity of these transporters, particularly under fungicide stress, remain insufficiently understood. Investigating these mechanisms is crucial for developing targeted strategies to mitigate fungicide resistance.

Fungi have additional mechanisms that contribute to drug resistance development in addition to these two well-studied types of multidrug transport proteins. Drug resistance caused by alterations in *ERG11*/*CYP51* (the target of azole fungicides) brought on by demethylation inhibitors (DMIs), such as gene mutations or overexpression, has been extensively documented. Studies have shown that *ERG11* upregulation is frequently linked to azole resistance in *Candida albicans*. Transcriptomic profiling of azole-resistant fungal strains has revealed differential expression of ERG-related genes and regulatory pathways, providing insights into resistance mechanisms in species such as *Cercosporidium personatum* and *Candida* spp. [20,21]. Fungal resistance to azole fungicides is also influenced by partial compensatory and catabolic mechanisms. For example, it has been demonstrated that fungal drug resistance is increased by mutations in *ERG3*, which encodes sterol D5,6-desaturase [22]. The cellular architecture of fungi is also essential for their fungicide resistance mechanisms. Fungal resistance is largely caused by ergosterol, chitin, and melanin, which are essential parts of fungal cell walls and membranes. For example, melanin can protect cells from chemical degradation by being deposited near cell walls, which are cross-linked by various macromolecules, thereby increasing cell strength and stiffness, altering cell permeability and cell swelling [23,24].

Pathogenic fungi, particularly *A. alternata*, have developed resistance mechanisms, such as efflux pump overexpression and alterations in ergosterol biosynthesis, which reduce the effectiveness of fungicides and enable survival under fungicidal stress. The objective of this study was to examine how *A. alternata* responded molecularly and physiologically to three fungicides: prochloraz (PCZ), fludioxonil (FLU), and mancozeb (MZ). The study assessed the effects of each fungicide at EC_50_, using physiological indicators and transcriptome data. The objectives were to find probable mechanisms underlying fungicide resistance in *A. alternata* and to gain a better understanding of its adaptive responses to fungicidal stress.

## 2. Materials and Methods

### 2.1. Activation and Sequencing of Fungal Pathogens

The *A. alternata* strain, AaNEFU1, used in this study was isolated by Xinbo Ma from Populus nigra leaves showing typical symptoms of infection on the campus of Northeast Forestry University (Harbin, Heilongjiang Province, China). Leaf samples were collected using sterile scissors and forceps, placed in sterile plastic bags, and immediately transported to the laboratory for processing. The infected tissues were surface-sterilized with 75% ethanol for 30 s, followed by 1% sodium hypochlorite for 2 min, and then rinsed 3 times with sterile distilled water. The sterilized tissues were plated onto potato dextrose agar (PDA, 200 g of potato, 16 g of agar, and 20 g of sucrose per liter of distilled water) medium and incubated at 25 ± 2 °C for 5–7 days. Single-spore isolation was performed to obtain a pure culture of *A. alternata*, which was identified based on morphological characteristics, ITS rDNA and β-tubulin sequencing [25]. To activate the strains that have been kept on PDA slant and in a refrigerator at 4 °C, they were inoculated onto PDA medium and cultivated for a week at 25 ± 2 °C. Mycelia were gathered after a successful activation, and the CTAB technique was used to extract the genomic DNA [26]. The isolated DNA was sent to Shanghai Personal Biotechnology Co., Ltd. (Shanghai, China) for genome frame sequencing after being snap-frozen in liquid nitrogen. The sequencing results were used as the reference genome for transcriptome sequencing.

### 2.2. Screening for Fungicide Sensitivity

Twelve widely used chemical fungicides with various modes of action were chosen based on the present usage of fungicides for treating foliar diseases in the Chinese market, as well as the principles of low toxicity, cost-effectiveness, and widespread application (Appendix A Table A1). The stock solutions of each fungicide were prepared according to the concentrations recommended in the instructions. The sensitivity of *A. alternata* isolates to fungicides was determined to estimate inhibition via a mycelial growth assay, a widely used in vitro antifungal susceptibility test [27]. The preserved pathogens were reactivated by culturing them on a PDA medium for 5 days. Following incubation, 5 mm diameter plugs were extracted from the actively growing margin of fungal colonies to ensure consistent inoculation. These plugs were placed centrally on PDA media supplemented with one of the following fungicide concentrations: 0.5 µg/mL, 5 µg/mL, 10 µg/mL, 50 µg/mL, and 100 µg/mL. The fungicide concentrations were selected based on previous studies on *Alternaria* spp. sensitivity, ensuring a broad range for 50% effective concentration (EC_50_) calculation and the regression equation [28,29]. This gradient allows for comparability with existing research and resistance assessment. Three replicates per concentration were employed in a completely randomized design to measure colony diameter and inhibition rate, with the controls consisting of PDA media with an equivalent volume of sterile water. Four fungicides with comparatively high sensitivity—difenoconazole, fludioxonil, mancozeb, and prochloraz—were chosen for further screening based on the findings of the preliminary screening. The selection of concentrations for the further screening was adjusted based on the results of the preliminary screening, while other steps remained the same as in the preliminary screening. The EC_50_ values and the regression equation of fungicide toxicity were computed using the findings of the additional screening.

### 2.3. Transcriptome Sequencing After Different Fungicide Treatments

Based on the results of the fungicide sensitivity screening, FLU, MZ, and PCZ were selected for subsequent experiments. AaNEFU1 was inoculated into a PDA medium that contained FLU, MZ, and PCZ at their corresponding EC_50_ values. The experiment followed a completely randomized design, with each treatment having three biological replicates. The control group (CK) substituted an equivalent volume of sterile water for the fungicides. The purpose of this experiment is to obtain raw materials for transcriptome sequencing. Fungal mycelia were scraped off the plates after 5 days of incubation at 25 °C, flash-frozen in liquid nitrogen, and then delivered to Beijing Biomarker Technologies Co., Ltd. for transcriptome sequencing and RNA extraction using the total RNA extraction kit (RNAsimple Total RNA Kit, Tiangen Biotech, Beijing, China; Cat. #. 4992858). The Illumina HiSeq2500 platform (Illumina Inc., San Diego, CA, USA) was used for sequencing. The cDNA libraries were prepared following the manufacturer’s protocol, and sequencing reads were quality-filtered before being mapped to the *A. alternata* reference genome (AaNEFU1) using HISAT2 (version 2.2.1) [30,31,32,33]. Differential expression analysis was conducted using DESeq2 (version 3.2.0), with differentially expressed genes identified based on a fold change of ≥2 and a false discovery rate (FDR) of <0.01.

### 2.4. Determination of Physiological Indicators

Tyrosinase activity, melanin content, malondialdehyde (MDA) content, superoxide dismutase (SOD) activity, catalase (CAT) activity, and soluble protein content in *A. alternata* were evaluated using the RNA-seq parallel samples. Melanin content was determined following the methodology described by Rudrappa [34], while the Coomassie Brilliant Blue reagent and the matching enzyme activity kits from Solarbio LIFE SCIENCES (Beijing, China) were used to measure the other physiological indicators [35,36,37]. Three biological replicates were used for each treatment. SPSS 25.0 software was used for statistical analysis, and one-way ANOVA (*p* < 0.05) was used to examine significant differences in the data.

## 3. Results

### 3.1. Fungicide Sensitivity Screening and EC_50_ Calculation

**Figure A1:**
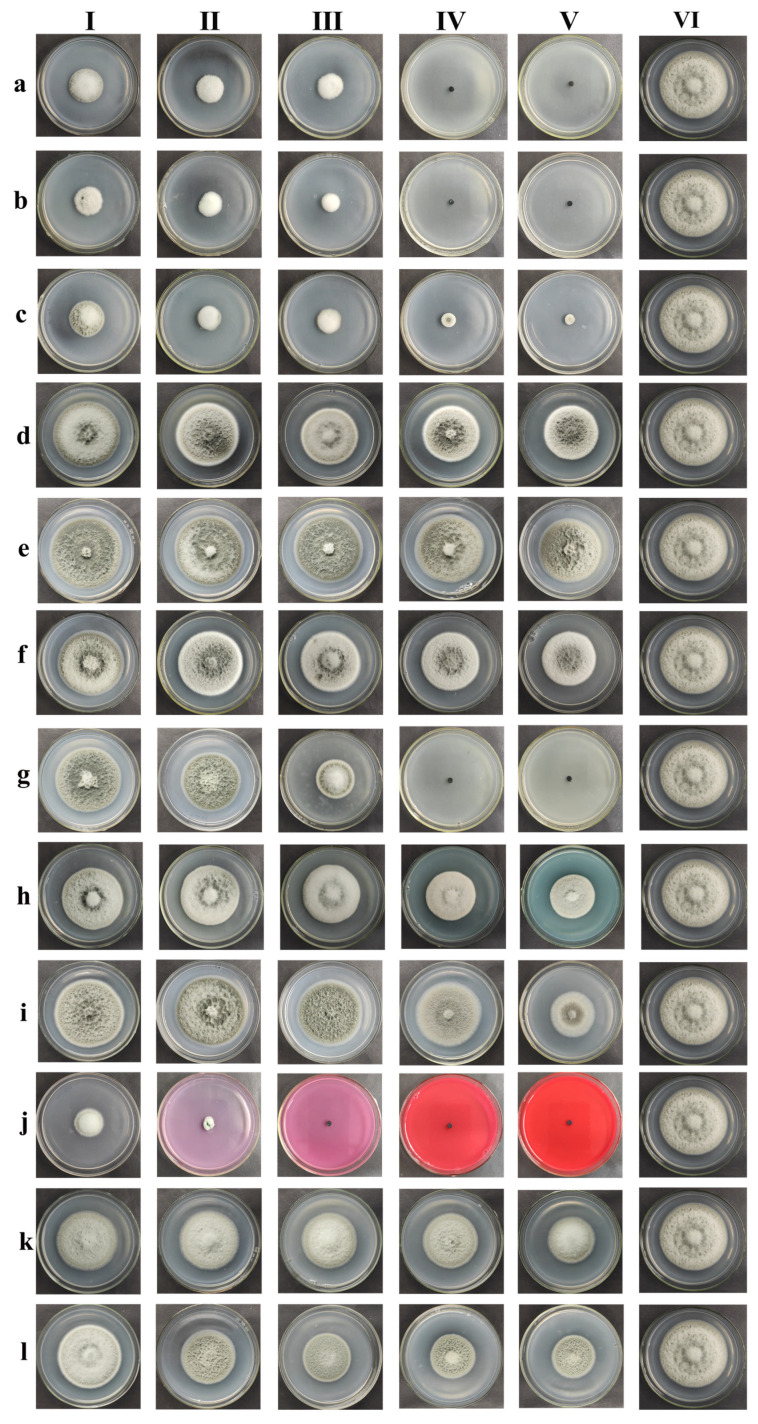
Preliminary screening results of *A. alternata* (AaNEFU1) mycelial growth to fungicides based on the mycelial growth inhibition rate. a–l represent difenoconazole, prochloraz, myclobutanil, carbendazim, thiophanate-Methyl, chlorothalonil, mancozeb, zineb, phosethy-Al, fludioxonil, azoxystrobin and triadimefon, respectively; I–VI represent 0.5 µg/mL, 5 µg/mL, 10 µg/mL, 50 µg/mL, 100 µg/mL, and CK, respectively.

**Figure A2:**
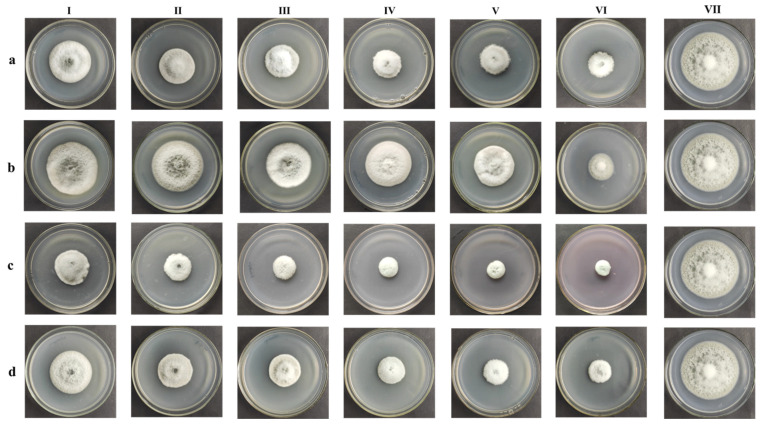
Sensitivity rescreening results of *A. alternata* (AaNEFU1) mycelial growth to fungicides based on the mycelial growth inhibition rate. a represents mancozeb (MZ), with I–VI representing 0.1, 0.5, 1, 5, 10, 15 μg/mL, respectively; b represents prochloraz (PCZ), with I–VI representing 0.1, 0.2, 0.4, 0.8, 2, 4 μg/mL, respectively; c represents fludioxonil (FLU), with I–VI representing 0.1, 0.2, 0.4, 0.8, 1, 2 μg/mL, respectively; d represents difenoconazole, with I–VI representing 0.1, 0.2, 0.4, 0.8, 2, 4 μg/mL, respectively; VII represents CK group.

The sensitivity screening results of AaNEFU1 against 12 fungicides are presented in Appendix A Table A2, Figure A1. FLU, MZ, PCZ, and difenoconazole were the fungicides that showed the strongest antifungal activity, achieving 100% inhibition rates at 50 µg/mL and 100 µg/mL. The mechanisms of action of difenoconazole (a protective curative agent) and PCZ (a protective eradicate) are the same, and both are DMI fungicides. MZ acts as a multi-site protectant, whereas FLU belongs to the PP (Phenylpyrrole) class of protectants. These four very effective fungicides were selected for further screening, with concentrations modified in accordance with the findings of the initial screening, in order to identify the best fungicides with various modes of action. The toxicity regression equations for MZ, PCZ, FLU, and difenoconazole were created following further screening (Table 1, Appendix A, Figure A2). MZ, PCZ, FLU, and difenoconazole had computed EC_50_ values of 14.66, 2.24, 0.35, and 2.91 µg/mL, respectively (Table 2). In these equations, y represents the inhibition rate, x denotes the fungicide concentration, and K is the slope, which indicates the sensitivity of *A. alternata* to each fungicide. PCZ exhibits better antifungal efficacy than difenoconazole, as evidenced by its EC_50_ value being lower and its toxicity regression equation slope (K value) being higher. Therefore, PCZ was identified as the most effective DMI fungicide in this experiment. Ultimately, three fungicides with superior antifungal activity and distinct mechanisms were selected for subsequent transcriptomic and other analyses and ranked according to their inhibitory effects: fludioxonil (EC_50_ = 0.35 µg/mL, K = 0.1686), prochloraz (EC_50_ = 2.24 µg/mL, K = 0.0723), and mancozeb (EC_50_ = 14.66 µg/mL, K = 0.0290).

### 3.2. Transcriptome Sequencing and Assembly

After Illumina sequencing, the transcriptome cDNA libraries from the 12 samples in the FLU, PCZ, MZ, and CK groups contained 75.13 GB of clean data. At least 5.88 GB of clean data was generated by each sample, and each sample’s Q30 score was higher than 90%, indicating high sequencing quality. All sample’s clean reads were aligned to the specified reference genome, AaNEFU1, with alignment efficiency over 95% in every group. Based on the alignment results, analyses for alternative splicing prediction, gene structure optimization, and the discovery of new genes were conducted, resulting in the identification of 1961 novel genes, 861 of which received functional annotations. The GC percentage for each sample surpassed 54%, and the Q30 percentage for each sample exceeded 94%, confirming that the sequencing quality was acceptable (Table 3). The number of reads aligned to the reference genome and the percentage of reads within the clean reads were no less than 96% (Table 4), establishing a foundation for subsequent differential gene analysis.

### 3.3. Identification and Analysis of Differentially Expressed Genes (DEGs)

The FDR threshold of 0.01 (i.e., corrected *p*-value < 0.01) and an absolute log2 fold change (log_2_FC) value of ≥1 was employed as cutoff criteria to identify DEGs across various libraries, specifically CK vs. FLU, CK vs. MZ, and CK vs. PCZ. A total of 2939 DEGs (1141 upregulated and 1798 downregulated) were identified between CK and FLU, representing the drug-responsive genes under FLU treatment; 744 DEGs (159 upregulated and 585 downregulated) were identified between CK and MZ, representing the drug-responsive genes under MZ treatment; and 1028 DEGs (312 upregulated and 716 downregulated) were identified between CK and PCZ, representing the drug-responsive genes under PCZ treatment.

**Figure 1:**
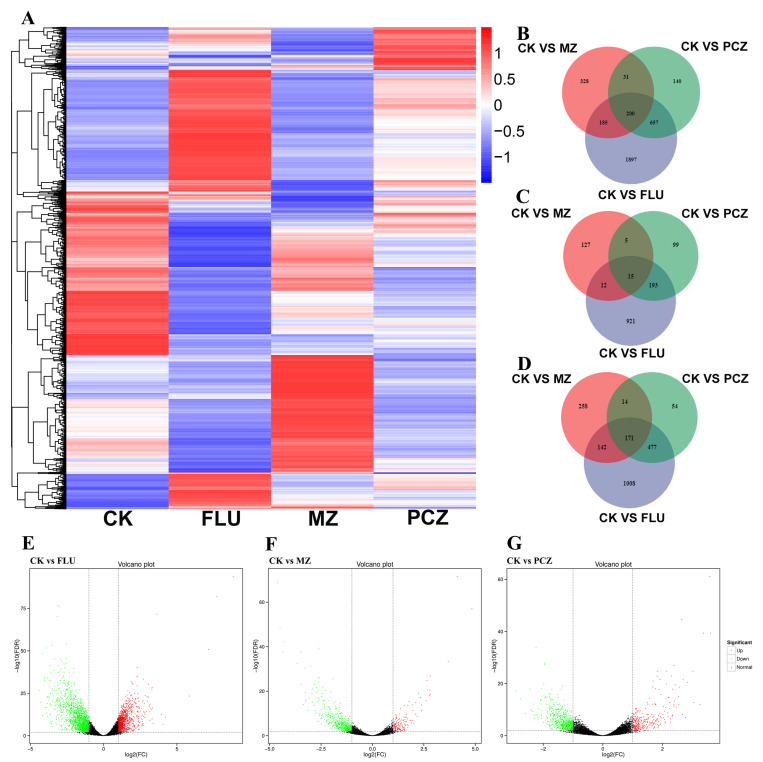
DEGs in *A. alternata* (AaNEFU1) under different fungicide treatment. (**A**) Heatmap cluster analysis of up- and downregulated genes per fungicide treatment. (**B**–**D**) Venn diagrams of the number of all, upregulated and downregulated DEGs between CK vs. FLU (blue), CK vs. MZ (red), and CK vs. PCZ (green). (**E**–**G**) Volcano diagrams of log2 fold changes in DEGs in response to FLU, MZ, and PCZ treatments, respectively. The dots represent genes that were upregulated (red) or downregulated (green).

The DEG profiles between CK, FLU, MZ, and PCZ treatments were visualized using hierarchical cluster (i.e., heatmap) analysis based on the Fragments Per Kilobase of exon model per Million mapped fragments (FPKM) values mentioned previously (Figure 1A). The results showed that *A. alternata* treated with FLU exhibited the highest number of DEGs, followed by PCZ and MZ treatments, indicating that a greater number of DEGs were involved in the response of *A. alternata* to FLU treatment. DEGs with similar expression trends were observed between the PCZ and FLU treatment groups; however, compared to FLU treatment, the fold changes for the majority of DEGs following PCZ treatment were smaller. Interestingly, the MZ treatment resulted in distinct expression patterns for most DEGs compared with both the FLU and PCZ treatments, which illustrates the unique feedback situation of *A. alternata* to the MZ treatment.

The distribution of all DEGs and upregulated DEGs between CK and FLU, CK and MZ, and CK and PCZ was demonstrated by means of Venn diagrams (Figure 1B–D) and the volcano plot analysis (Figure 1E,F). Notably, the CK vs. FLU group showed a wider range of fold change (FC) values, both upregulated and downregulated, when compared to the other fungicide treatment groups. The Venn diagrams also showed that the overlapping region of the CK vs. FLU, CK vs. MZ, and CK vs. PCZ groups contained 200 DEGs, of which 15 overlapped in the upregulated category. The three Venn diagrams showed that, regardless of the total number of DEGs, the upregulated and downregulated DEGs, and the number of unique DEGs were most frequent in the FLU treatment. This observation aligns with earlier statistical analyses of DEGs and heatmap results.

In conclusion, these results based on DEG analysis indicate that the response of *A. alternata* DEGs to FLU treatment is the most intense, with similarities in DEG expression between PCZ treatment and FLU treatment, and with unique DEG expression patterns observed after MZ treatment.

### 3.4. GO and KEGG Enrichment Analysis of DEGs

**Figure 2:**
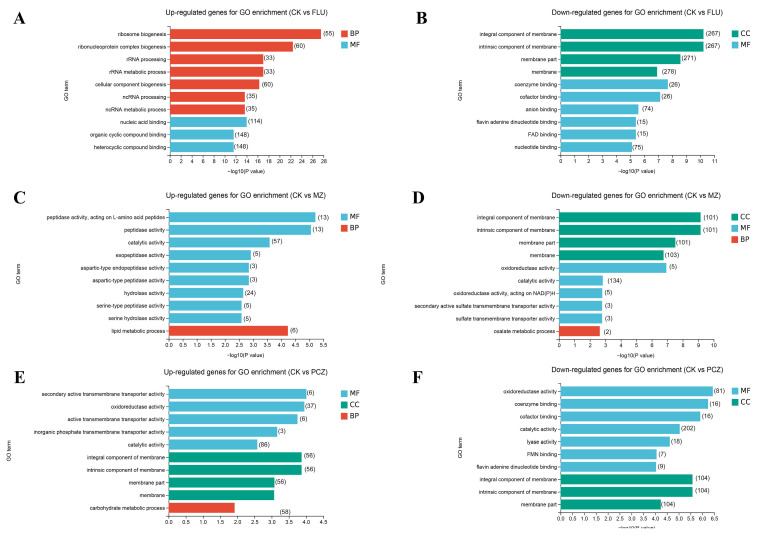
GO enrichment analysis of DEGs under different fungicide treatments. (**A**,**B**) The top 10 most significantly up- and downregulated GO categories following FLU treatment. (**C**,**D**) The top 10 most significantly up- and downregulated GO categories following MZ treatment. (**E**,**F**) The top 10 most significantly up- and downregulated GO categories following PCZ treatment. The length of columns indicates a significant degree of genes enriched in given GO items, and the number inside the parentheses represents the number of enriched genes; BP (red), CC (green), and MF (blue) represent the abbreviations of Biological Process, Cellular Component and Molecular Function, respectively.

By conducting a Gene Ontology (GO) enrichment analysis of the DEGs, the top ten most significantly enriched categories were chosen for each group to comprehend the functions of the DEGs (Figure 2). The outcomes of the GO enrichment analyses differed depending on the fungicide treatments.

Among the upregulated GO enrichment terms related to Biological Processes (BPs), the FLU treatment was associated primarily with ribosome biogenesis, rRNA processing, ncRNA processing, and cellular component biogenesis (Figure 2A). In contrast, the results of the MZ treatment were predominantly related to lipid metabolic processes, whereas those of the PCZ treatment were mainly linked to carbohydrate metabolic processes (Figure 2C–E). Conversely, among the GO enrichment terms for BP that were found to be downregulated, only the MZ treatment was associated with the oxalate metabolic process (Figure 2D). No GO enrichment terms were found to be enriched for the other two fungicide treatments (Figure 2B,F).

In the upregulated GO enrichment terms related to Cellular Component (CC), only the PCZ treatment showed enrichment associated with components of the membrane, while no GO terms were enriched in the other fungicide treatment (Figure 2A,C,E). Among the downregulated GO enrichment terms related to CC, all three treatment groups were associated with components of membranes (Figure 2B,D,F).

For Molecular Function (MF) in the upregulated GO enrichment terms, FLU treatment was associated with nucleic acid binding, organic cyclic compound binding, and heterocyclic compound binding (Figure 2A); MZ treatment was related to peptidase and hydrolase activities (Figure 2C); PCZ treatment was linked to transmembrane transporter activities, oxidoreductase activity, and catalytic activity (Figure 2E). In the downregulated MF GO enrichment terms, FLU treatment was associated with mainly coenzyme binding, cofactor binding, anion binding, flavin adenine dinucleotide (FAD) binding, and nucleotide binding (Figure 2B); MZ treatment was linked to oxidoreductase activity, catalytic activity, and transmembrane transporter activities (Figure 2D); PCZ treatment was associated with oxidoreductase activity, coenzyme binding, cofactor binding, catalytic activity, lyase activity, FMN binding and flavin adenine dinucleotide binding (Figure 2F).

**Figure 3:**
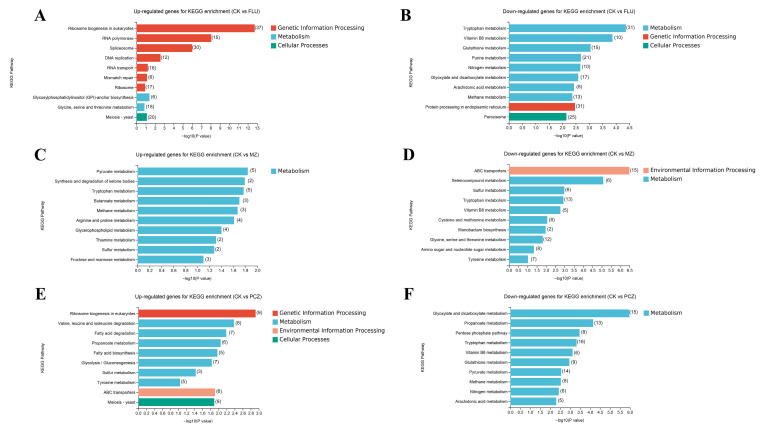
KEGG enrichment analysis of DEGs after treatment with different fungicides. (**A**,**B**) The top 10 most significantly upregulated and downregulated categories after FLU treatment. (**C**,**D**) The top 10 most significantly upregulated and downregulated categories after MZ treatment. (**E**,**F**) The top 10 most significantly upregulated and downregulated categories after PCZ treatment. The length of columns indicates a significant degree of genes enriched in given KEGG pathways, and the number inside the parentheses represents the number of enriched genes; red, green, blue, and yellow stripes represent genetic information processing, Cellular Processes, metabolism, and environmental information processing, respectively.

The top 10 most significantly enriched pathways for each treatment were selected based on Kyoto Encyclopedia of Genes and Genomes (KEGG) pathway enrichment analysis. The results revealed that pathways associated with genetic information processing, cellular processes, metabolism, and environmental information processing were highly enriched among the DEGs in fungicide-treated *A. alternata* (Figure 3).

Among the upregulated pathways related to genetic information processing, FLU treatment was associated with ribosome biogenesis in eukaryotes, RNA polymerase, spliceosome, DNA replication, RNA transport, mismatch repair, and ribosome (Figure 3A). PCZ treatment was primarily associated primarily with ribosome biogenesis in eukaryotes (Figure 3E). No DEGs were enriched in genetic information processing after MZ treatment (Figure 3C). Regarding downregulated DEGs associated with genetic information processing, FLU treatment was enriched in protein processing in endoplasmic reticulum, whereas no enrichment in genetic information processing pathways was observed for PCZ and MZ treatments (Figure 3B,D,F).

For upregulated DEGs related to cellular processes, FLU treatment was associated with the meiosis-yeast, a trend also observed with PCZ treatment (Figure 3A,E). However, MZ treatment showed no DEGs linked to cellular process pathways (Figure 3C). Regarding downregulated DEGs associated with cellular processes, FLU treatment was enriched in the peroxisome, whereas no enrichment in cellular process pathways was observed for PCZ or MZ treatments (Figure 3B,D,F).

The upregulated KEGG pathways associated with metabolism exhibited distinct patterns under different fungicide treatments. FLU treatment primarily affected glycosylphosphatidylinositol (GPI)-anchor biosynthesis and glycine, serine, and threonine metabolism (Figure 3A), suggesting a potential disruption in membrane protein anchoring and amino acid utilization. In contrast, MZ treatment triggered a broader metabolic response, with significant changes in pathways related to energy metabolism (pyruvate metabolism, synthesis and degradation of ketone bodies, butanoate metabolism), amino acid catabolism (tryptophan metabolism, arginine and proline metabolism), and cellular homeostasis (methane metabolism, glycerophospholipid metabolism, thiamine metabolism, sulfur metabolism, and fructose and mannose metabolism) (Figure 3B). PCZ treatment was mainly associated with pathways such as valine, leucine, and isoleucine degradation, fatty acid degradation, propanoate metabolism, fatty acid biosynthesis, glycolysis/gluconeogenesis, sulfur metabolism, and tyrosine metabolism (Figure 3E). Among the downregulated KEGG pathways associated with metabolism, FLU treatment was associated primarily with tryptophan metabolism, vitamin B6 metabolism, glutathione metabolism, purine metabolism, nitrogen metabolism, glyoxylate and dicarboxylate metabolism, arachidonic acid metabolism, and methane metabolism (Figure 3B). MZ treatment mainly involves selenocompound metabolism, sulfur metabolism, tryptophan metabolism, vitamin B6 metabolism, cysteine and methionine metabolism, monobactam biosynthesis, glycine, serine and threonine metabolism, amino sugar and nucleotide sugar metabolism and tyrosine metabolism (Figure 3D). PCZ treatment was associated primarily with glyoxylate and dicarboxylate metabolism, propanoate metabolism, pentose phosphate pathway, tryptophan metabolism, vitamin B6 metabolism, glutathione metabolism, pyruvate metabolism, methane metabolism, nitrogen metabolism, arachidonic acid metabolism (Figure 3F).

Among the upregulated KEGG pathways associated with the environmental information processing pathways, only the DEGs related to ABC transporters were enriched after PCZ treatment (Figure 3E). Conversely, in the downregulated environmental information processing pathways, only the DEGs related to ABC transporters were enriched after MZ treatment (Figure 3D).

All the KEGG-enriched DEGs, as components of metabolic or signal-transduction pathways, were well coincident with the results of GO enrichment. From the results of GO and KEGG enrichment analysis, the FLU treatment group and the PCZ treatment group had some of the same enrichment categories and pathways. However, the MZ treatment group rarely had the same categories and pathways as the other two groups. This is consistent with the previous analysis results.

### 3.5. Collinear Trend Analysis Based on DEGs

DEGs were subjected to a collinear trend analysis in order to better understand how three fungicides with varied modes of action affected the gene expression patterns of *A. alternata* under stress. Genes with FPKM values greater than one were considered expressed. Collinear trend analysis and its top ten GO enrichment analysis entries showed that the expression trends of genes varied among fungicide treatment groups.

**Figure 4:**
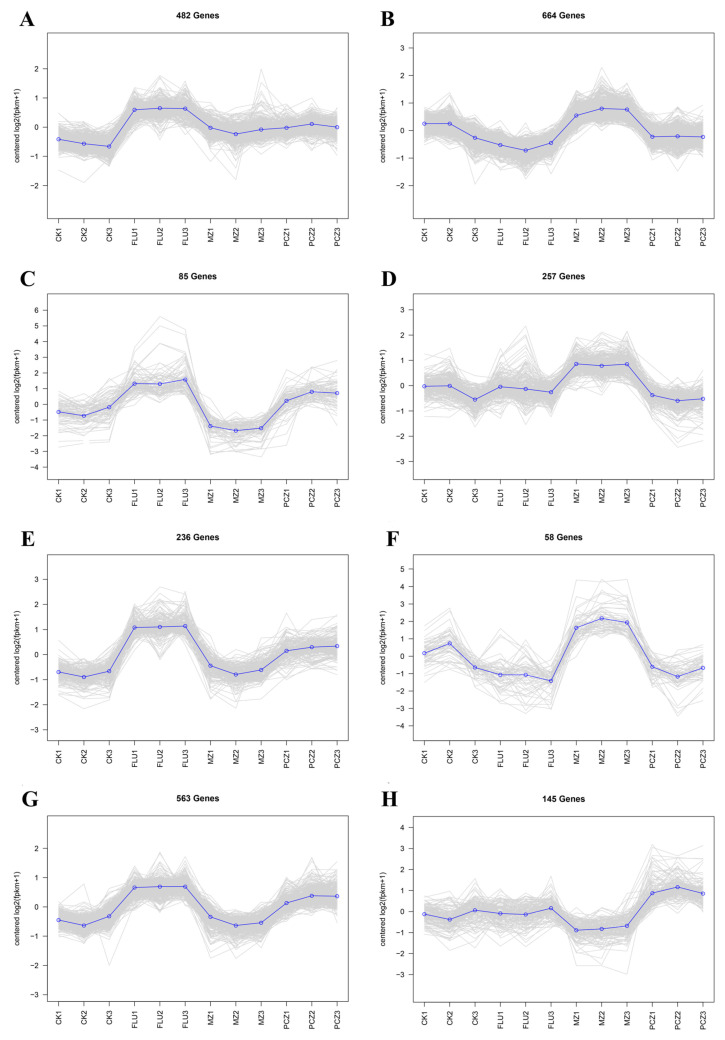
Collinear trend analysis of DEGs after treatment with different fungicides. The gray lines in the background represent the expression trends of individual genes within the cluster, while the blue line indicates the average trend of all genes after normalization. (**A**) Total of 482 genes with an upregulation trend after treatment with all three fungicides. (**C**,**E**,**G**) Total of 884 genes with an upregulation trend after treatment with FLU and PCZ. (**B**,**D**,**F**) Total of 979 genes with an upregulation trend after treatment with MZ. (**H**) Total of 145 genes with an upregulation trend after treatment with PCZ.

**Figure 5:**
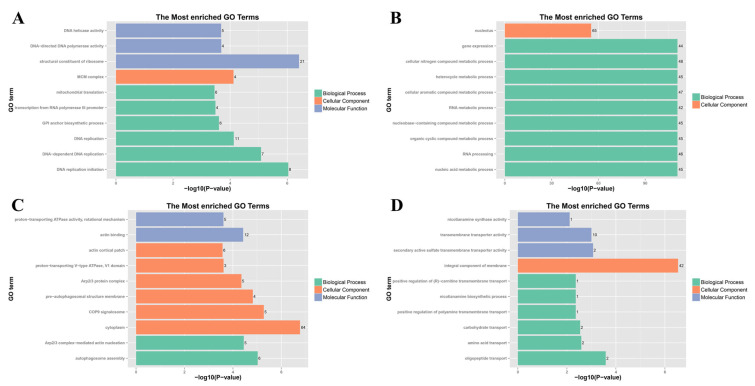
GO enrichment analysis of DEGs used for collinear trend analysis after treatment with different fungicides. (**A**) The top 10 most significantly upregulated categories after FLU, MZ, and PCZ treatment. (**B**) The top 10 most significantly upregulated categories after FLU and PCZ treatment. (**C**) The top 10 most significantly upregulated after MZ treatment. (**D**) The top 10 most significantly upregulated after PCZ treatment. The length of columns indicates a significant degree of genes enriched in given GO items, and the number inside the parentheses represents the number of enriched genes; green, orange, and blue represent the abbreviations of Biological Processes, Cellular Components, and Molecular Function, respectively.

Under FLU treatment, the highest levels of gene expression were noted. Across all three fungicide treatments, 482 genes showed a consistently higher expression pattern, as shown in Figure 4A. GO enrichment analysis of these genes indicated that the top ten enriched categories were primarily associated with ribosomal structure and DNA replication (Figure 5A). It was found that 884 genes had a consistently elevated trend in both the FLU and PCZ treatments, with greater expression levels in the FLU treatment (Figure 4C,E,G). GO enrichment analysis of these genes revealed that the top ten enriched categories were largely associated with nucleic acid metabolism and RNA processing (Figure 5B). On the other hand, 979 genes showed a clear tendency of increased expression only when treated with MZ (Figure 4B,D,F). The top ten most highly enriched categories, according to GO enrichment analysis, were primarily associated with actin and autophagosomes (Figure 5C). Additionally, under PCZ treatment, 145 genes showed a tendency of exclusively enhanced expression (Figure 4H). GO enrichment analysis revealed that the top ten most significantly enriched categories were primarily associated with transmembrane transport of different substances and the biosynthesis of nicotinamide correlation (Figure 5D).

In conclusion, some common upregulated genes appeared under different fungicide treatments, but more specific upregulated genes were also seen. The most evident of them was the pattern of elevated genes during FLU treatment. The genes whose expression was upregulated in the FLU treatment and PCZ treatment groups were similar. In contrast, the genes whose expression was upregulated in the MZ treatment group were more unique. This result resonates with the analysis of DEGs, as well as the annotation analysis results of GO and KEGG databases. These findings imply the possible presence of cross-resistance mechanisms in *A. alternata* to FLU and PCZ and are consistent with DEG studies and GO/KEGG annotation results.

### 3.6. Analysis of Fungicide Resistance-Related Genes Based on DEGs

**Figure 6:**
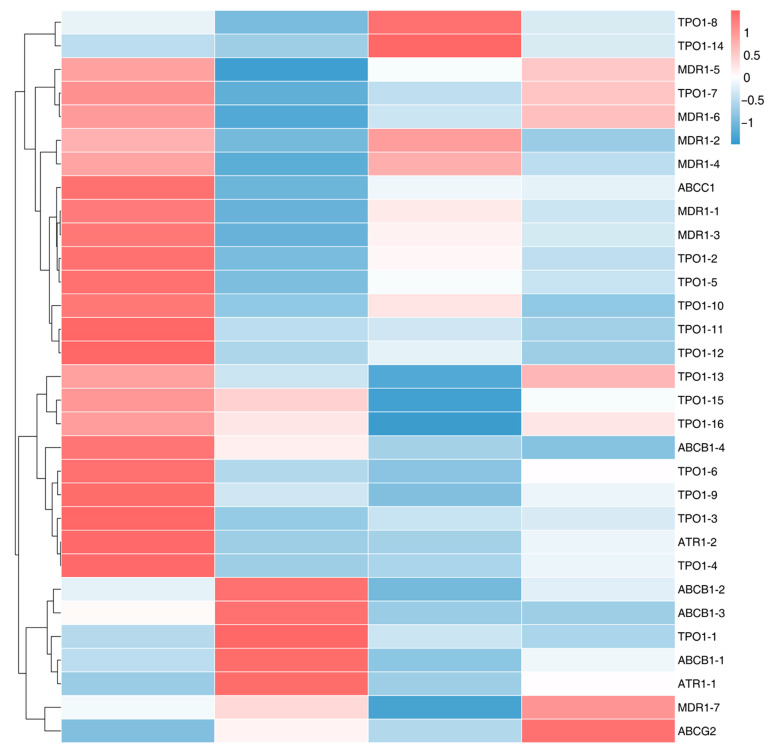
Heatmap cluster analysis of up- and downregulated genes of ABC and MFS transporter proteins under each fungicide treatment.

On the basis of the KEGG annotation of DEGs following treatment with three different fungicides, 31 MDR genes were identified in *A. alternata*. These MDR genes included ABC transporter and MFS transporter. Specifically, 6 ABC transporter genes belonging to subfamilies B, C, and G were identified, and 25 MFS transporter genes were classified within the DHA1 and DHA2 families (Table 5). A heatmap of these genes’ FPKM values revealed that most genes were downregulated following fungicide treatment, with only a tiny subset upregulated following fungicide treatment (Figure 6). Among these genes, the most upregulated genes were seen after FLU treatment. This indicates that after FLU treatment, the MDR upregulation response in *A. alternata* is the most intense, followed by treatments with MZ and PCZ, which is also related to the highest fungicide sensitivity of *A. alternata* to FLU.

**Figure 7:**
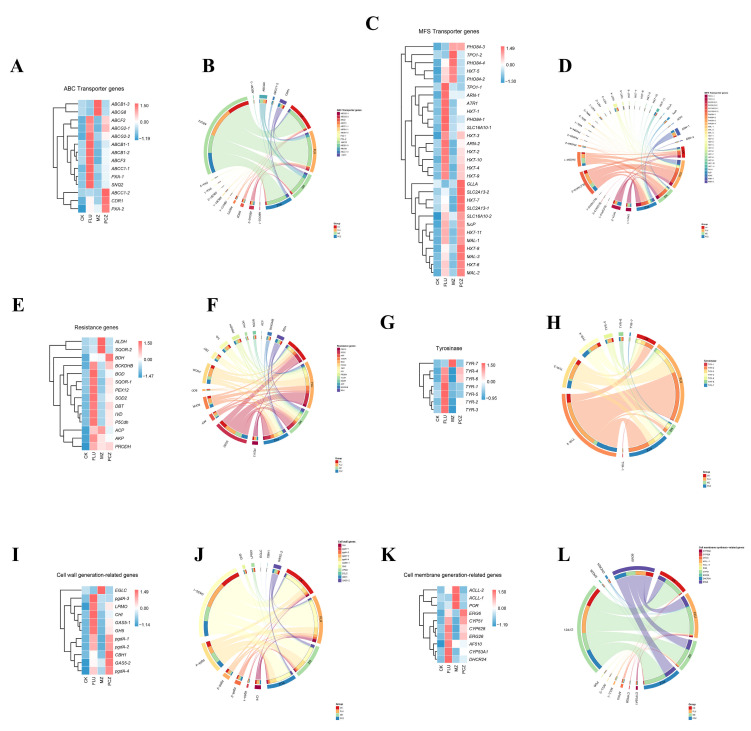
Analysis of 88 upregulated DEGs related to fungal resistance identified based on annotations from the KEGG database and previous research findings. (**A**,**B**) Heatmap cluster and chord diagram analysis of upregulated genes of ABC transporter genes under each fungicide treatment. (**C**,**D**) Heatmap cluster and chord diagram analysis of upregulated genes of MFS transporter genes under each fungicide treatment. (**E**,**F**) Heatmap cluster and chord diagram analysis of upregulated genes of resistance genes under each fungicide treatment. (**G**,**H**) Heatmap cluster and chord diagram analysis of upregulated genes of tyrosinase genes under each fungicide treatment. (**I**,**J**) Heatmap cluster and chord diagram analysis of upregulated genes of cell wall biosynthesis-related genes under each fungicide treatment. (**K**,**L**) Heatmap cluster and chord diagram analysis of upregulated genes of cell membrane biosynthesis-related genes under each fungicide treatment.

The majority of MDR genes were downregulated, according to the KEGG database annotation. This suggests that most MDR genes were suppressed following fungicide treatment, with just a few MDR genes upregulated in response to the fungicide treatment. The investigation of fungicide resistance genes is still incomplete because the majority of research has concentrated on ABC transporters and MFS transporters. Based on the KEGG database annotations and prior studies, 88 DEGs linked to fungal fungicide resistance were found in order to better explore the possible fungicide resistance mechanisms in *A. alternata*. These DEGs were classified into six categories: ABC transporters (14 genes), MFS transporters (29 genes), resistance genes (14 genes), tyrosinases (10 genes), cell wall biosynthesis-related genes (11 genes), and cell membrane biosynthesis-related genes (10 genes). To illustrate these DEGs’ expression changes and examine the FPKM variances among the four treatment groups, an interactive chord diagram and heatmap were created using their FPKM values (Figure 7).

The majority of the ABC transporter genes were found to be significantly expressed in FLU treatment, and the FPKM of those genes was likewise higher in PCZ treatment than in CK and MZ treatment, according to this study. *ABCC1-2*, *CDR1*, and *PXA-2* are three more genes that are substantially expressed during PCZ treatment. Additionally, the FPKM of *PXA-2* and *CDR1* in the FLU treatment was higher than those in the other two treatments. *ABCB1-3* and *ABCG8* are the two distinct, significantly expressed genes in MZ treatment. While the total FPKMs of the FLU treatment genes were above those of the other treatments, *ABCF2* showed the highest advantage among all the genes (Figure 7A,B).

Among the MFS transporter genes, most genes were highly expressed in FLU treatment and PCZ treatment, and only five genes were highly expressed in MZ treatment. Some of the distinct genes that were highly expressed in each treatment were *TPO1-1*, *ARN-1*, *PHO84-1*, and *SLC16A10-1* of FLU treatment; *TPO1-2* of MZ treatment; *GLLA*, and *SLC2A13-2* of PCZ treatment. In terms of FPKM for all genes, *SLC16A10-1* and *PHO84-1* exhibited the most dominant, while the overall FPKMs of FLU treatment genes were greater than those of the other treatments (Figure 7C,D).

The majority of the genes in the resistance gene research were still highly expressed in both PCZ and FLU treatments, but neither treatment showed any distinct highly expressed genes. There are uniquely expressed genes, *ALDH* and *SQOR-2*, in the MZ treatment. *SOD2* had the most advantage when considering the FPKM of all genes; however, the total FPKM of the genes treated with FLU was higher than that of the other treatments (Figure 7E,F).

The analysis of tyrosinase-related genes revealed that all TYRs except for *TYR-7* were highly expressed in the FLU therapy, while *TYR-4* and *TYR-6* were highly expressed in the PCZ treatment and *TYR-7* was the only TYR that was strongly expressed in the MZ treatment. While the aggregate FPKM of the genes treated with FLU and PCZ was higher than that of the other treatments, *TYR-2* had the most benefit among all the genes’ FPKM values. Interestingly, the total gene expression in MZ treatment was even lower than that in CK treatment (Figure 7G,H).

In the study of cell wall generation-related genes, all genes except *EGLC* were highly expressed in FLU treatment, and *EGLC* was only highly expressed in MZ treatment. Among them, *pgdA-3* and *CHI* were the only genes that were uniquely highly expressed in the FLU treatment; there were no unique highly expressed genes in PCZ treatment. *GAS5-1* had the most advantage among all the genes’ FPKM values, and the overall FPKM of FLU treatment and PCZ treatment genes was greater than those of the other treatments (Figure 7I,J).

Research on genes associated with cell membrane biosynthesis indicated that *DHCR24* was uniquely expressed in FLU therapy, while *ACLL-1* and POR were substantially elevated solely in MZ treatment. No uniquely highly expressed genes were observed in the PCZ treatment. Based on the FPKM values of all genes, *CYP51* and *ERG6* exhibited the most significant expression levels, with the overall FPKM values of genes in the FLU treatment and PCZ treatment being higher than those in the other treatments (Figure 7K,L).

In conclusion, fungicide resistance mechanisms are similar for PCZ and FLU treatments, with FLU treatment exhibiting higher levels of fungicide resistance-related gene expression. Some distinct fungicide resistance genes are still present in the MZ treatment, despite the fact that the expression of genes linked to fungicide resistance is less active there than in the other two treatments.

### 3.7. Analysis of Fungicide Resistance-Related Pathways Based on DEGs

#### 3.7.1. Ergosterol Biosynthesis Pathway

**Figure 8:**
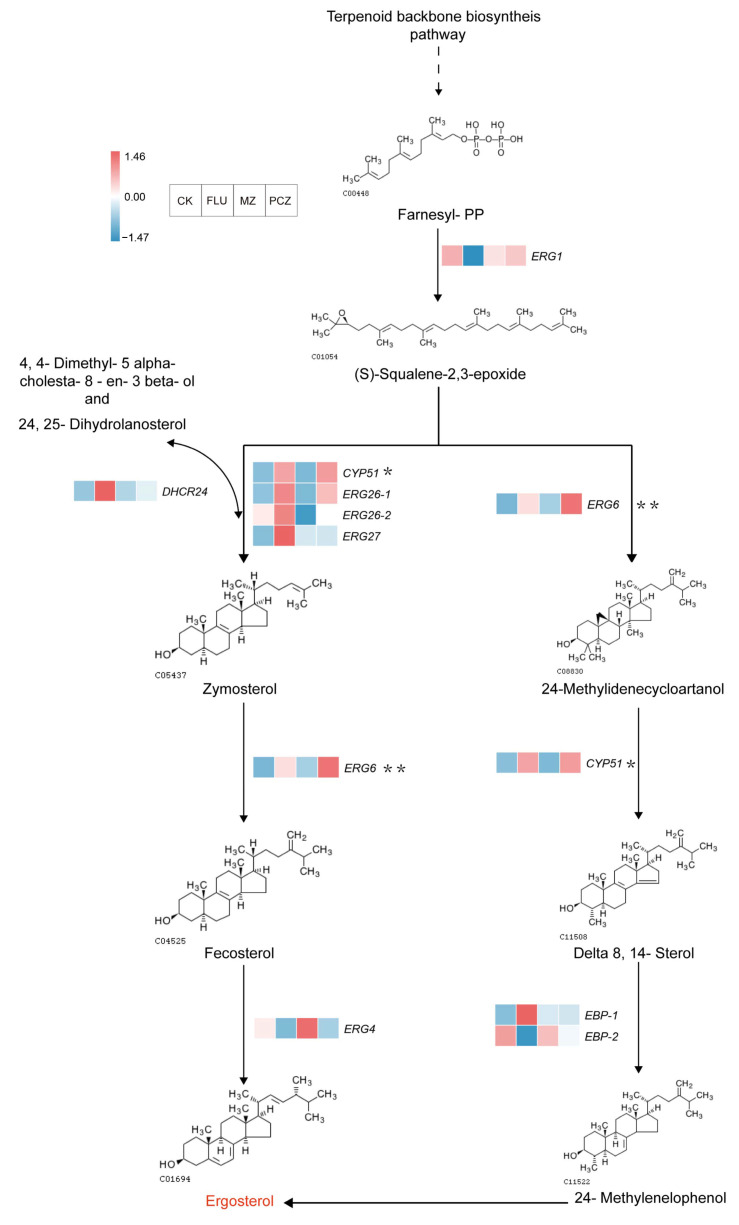
Ergosterol biosynthesis pathway of AaNEFU1 and the changes in DEGs. The direction of the arrows indicates the ergosterol synthesis direction. The heatmaps shown beside arrows denote the expression of the relevant DEGs in the different fungicide treatments, with the color related to the level of gene expression. The asterisk (*) indicates the key genes for the pathway. Double asterisks (**) indicate statistically significant differences in gene expression (*p* < 0.01).

Many studies have demonstrated the strong correlation between fungicide resistance and the underlying resistance mechanisms and ergosterol, a significant component of fungal cell membranes. The ergosterol biosynthesis pathway under three fungicide treatments was thoroughly analyzed based on the expression patterns of DEGs in the steroid biosynthesis pathway (ko00100) of the KEGG database (Figure 8). Six DEGs were elevated after FLU therapy, three DEGs after PCZ treatment, and no DEGs after MZ treatment in relation to ergosterol synthesis. The DEG expression profiles linked to the synthesis of ergosterol were used to identify two biosynthetic pathways.

The first pathway involves the synthesis of farnesyl-PP through the terpenoid backbone biosynthesis pathway. Farnesyl-PP is then converted into (S)-squalene-2,3-epoxide under the regulation of squalene monooxygenase (*ERG1*). Subsequently, (S)-squalene-2,3-epoxide is converted into zymosterol through the regulation of *DHCR24*, *CYP51*, *ERG26-1*, *ERG26-2*, and *ERG27*. Zymosterol is then converted into fecosterol under the regulation of *ERG6*, and finally, fecosterol is transformed into ergosterol under the regulation of *ERG4*.

The other ergosterol biosynthesis pathway begins with the conversion of (S)-squalene-2,3-epoxide into 24-methylidenecycloartanol under the regulation of *ERG6*. This is followed by the formation of Delta 8,14-sterol through the action of *CYP51*, and then, under the regulation of *EBP-1* and *EBP-2*, 24-methylenelophenol is generated. Finally, ergosterol is synthesized. In this entire ergosterol biosynthesis pathway, a total of nine DEGs were identified following FLU treatment, consisting of six upregulated DEGs and three downregulated DEGs. After MZ treatment, only one downregulated DEG was found, while three upregulated DEGs were observed following PCZ treatment (Table 6). These findings indicate that the response of the ergosterol biosynthesis pathway is most pronounced following FLU treatment, followed by PCZ, and least after MZ treatment. This study is the first to successfully model the two synthesis pathways of ergosterol in *A. alternata*, which is significant because ergosterol is a major component of fungal cell membranes and contributes to fungicide resistance. This helps to shed light on the mechanisms behind fungal fungicide resistance.

This research shown that the genes *CYP51* and *ERG6* are often regulated, especially in response to PCZ and FLU treatments. CK vs. FLU (log_2_FC = 1.04) and CK vs. PCZ (log_2_FC = 1.55) for *ERG6* and CK vs. FLU (log_2_FC = 1.02) and CK vs. PCZ (log_2_FC = 1.05) for *CYP51* were the expression fold changes that were noted. The downregulation of *ERG4* in the last stage of ergosterol production following FLU and PCZ treatments may be partially explained by the overexpression of *ERG6*, which may activate alternative pathways and cause the accumulation of toxic dienols rather than ergosterol. According to studies on fungal diseases, point mutations in *ERG11*/*CYP51*, as well as alterations in the promoter region or mutations in transcription factors that cause *ERG11*/*CYP51* overexpression and decrease azole sensitivity, are the most frequent resistance mechanisms to azole antifungals. Interestingly, *CYP51* remained elevated after treatment, despite being the target site of PCZ. This suggests that *CYP51* could be a resistance gene for *A. alternata* to PCZ or DMI fungicides.

From the overall ergosterol biosynthesis pathway, the largest number of upregulated DEGs were observed after FLU treatment. Additionally, FLU and PCZ treatments shared three upregulated genes, *ERG26-1*, *ERG6*, and *CYP51*, while no DEGs were upregulated in this pathway following MZ treatment. Moreover, most genes in the MZ treatment showed decreased expression, but the expression of *ERG4*, a key gene involved in the regulation of fecosterol conversion to ergosterol, was only increased after MZ treatment. This suggests that the gene expression profile following MZ treatment is distinct from the other two fungicides, and this result is consistent with the findings from the co-expression trend analysis.

#### 3.7.2. Melanin Biosynthesis Pathway

The DOPA and DHN melanin biosynthesis pathways are currently the two main processes for fungal melanin production, and the model organisms for both pathways are *Aspergillus fumigatus* and *Cryptococcus neoformans*, respectively [38].

**Figure 9:**
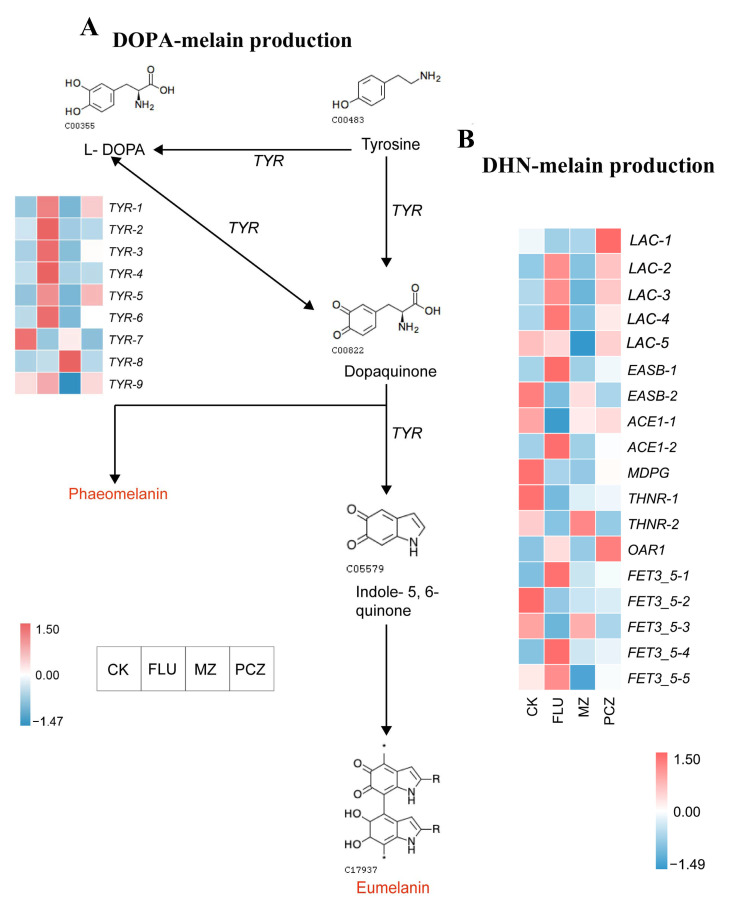
Melanin biosynthesis pathway and DEG changes in AaNEFU1. (**A**) DOPA melanin biosynthetic pathway. The direction of the arrows indicates the melanin synthesis direction. The heatmaps shown beside arrows denote the expression of the relevant DEGs in the different fungicide treatments, with the color related to the level of gene expression. (**B**) Heatmap for expression of DEGs involved in the DHN melanin biosynthesis pathway.

The DOPA melanin biosynthetic pathway begins with either Tyrosine or L-dopamine (L-DOPA) (Figure 9A). If Tyrosine serves as the precursor, it is first converted to L-DOPA and subsequently to Dopaquinone. When L-DOPA is the precursor, it directly converts into Dopaquinone. Dopaquinone is then transformed into Indole-5,6-quinone, which is ultimately converted into melanin, including Eumelanin and Phaeomelanin. The activity of tyrosinase regulates this entire biosynthetic pathway. Regarding tyrosinase genes expression, six tyrosinase genes were upregulated under FLU treatment, two under PCZ treatment, and only one under MZ treatment. Among these, four tyrosinase genes (*TYR-1*, *TYR-2*, *TYR-4*, and *TYR-6*) were exclusively upregulated in the FLU treatment. The uniquely upregulated tyrosinase in the MZ treatment was *TYR-8*, while no uniquely upregulated tyrosinase was observed under PCZ treatment (Table 7). This suggests that tyrosinase plays a key role in regulating the DOPA melanin biosynthetic pathway in *A. alternata*. The number of upregulated response genes was highest after FLU treatment, followed by PCZ treatment, and the lowest after MZ treatment. Notably, the two tyrosinase genes upregulated under PCZ treatment were also upregulated in the FLU treatment. With only one tyrosinase gene upregulated, the upregulated gene expression after MZ treatment, in contrast to the upregulated genes in the other two treatment groups, showed distinctive features.

The DHN melanin biosynthesis pathway begins with Malonyl-CoA, which induces the polymerization of 1,8-dihydroxynaphthalene (DHN), leading to the production of DHN melanin. Genes related to DHN melanin production include laccase, polyketide synthase, tetrahydroxynaphthalene reductase, and multicopper oxidase. Under FLU treatment, three laccase genes, two polyketide synthase genes, and two multicopper oxidase genes were upregulated, while one laccase gene, three polyketide synthase genes, two tetrahydroxynaphthalene reductase genes, and two multicopper oxidase genes were downregulated. Only DEGs associated with DHN melanin biosynthesis, such as two laccase genes, one polyketide synthase gene, and three multicopper oxidase genes, were downregulated following MZ treatment; in contrast, one tetrahydroxynaphthalene reductase gene was upregulated and one laccase gene, one polyketide synthase gene, one tetrahydroxynaphthalene reductase gene, and two multicopper oxidase genes were downregulated following PCZ treatment (Figure 9B). Regarding unique upregulated genes, FLU treatment elicited the strongest response among DHN melanin biosynthesis-related genes, including *LAC-2*, *LAC-3*, *LAC-4*, *EASB-1*, *ACE1-2*, *FET3_5-1*, and *FET3_5-4*. Meanwhile, PCZ treatment induced a unique upregulated DEG, *OAR1* (Table 7).

This work successfully simulated the melanin biosynthesis process of *A. alternata* for the first time using the model organisms’ melanin biosynthesis pathways as a template. By clarifying the regulatory processes behind melanin formation in *A. alternata*, it provided a theoretical basis for examining possible fungicide resistance mechanisms in related fungal species.

### 3.8. Correlation Between Some Physiological Indexes of A. alternata and Potential Fungicide Resistance-Related Genes

**Figure 10:**
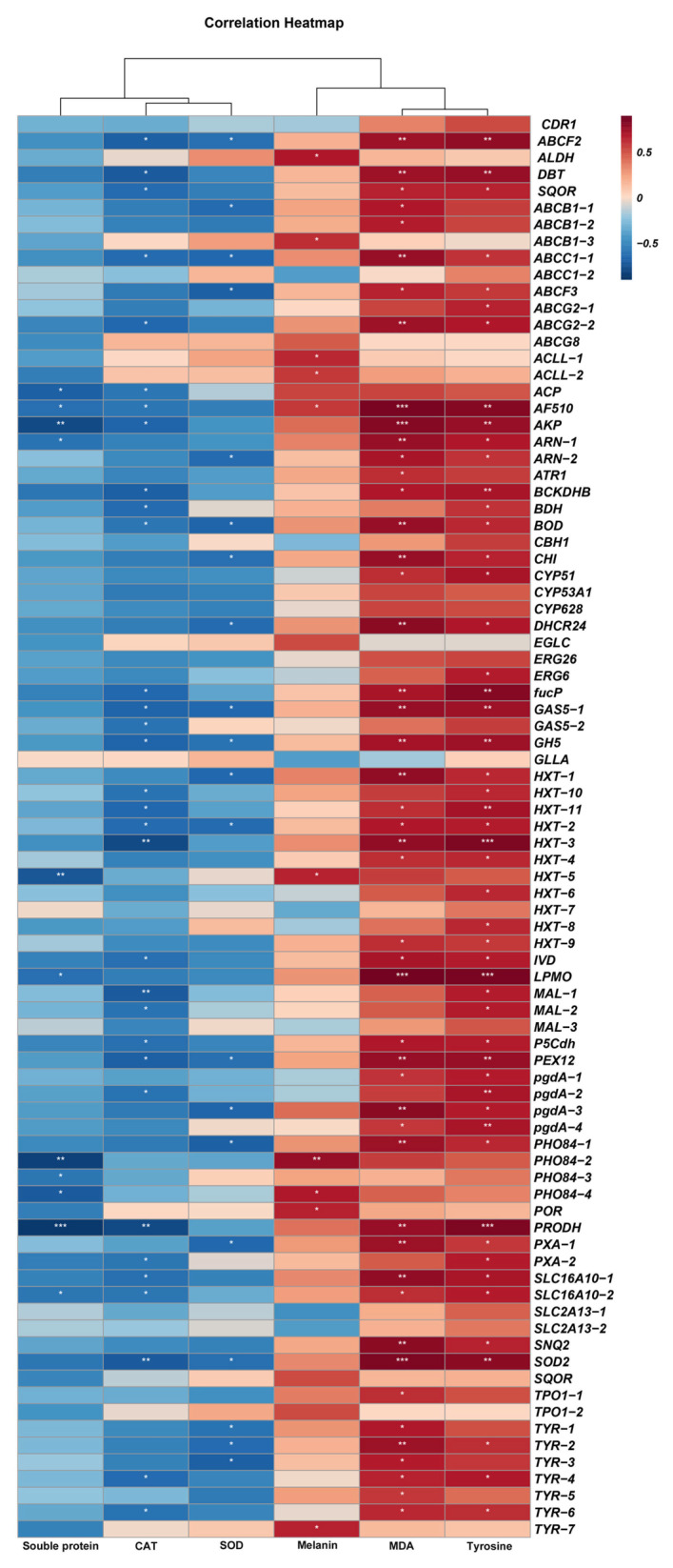
Heatmap of correlation between DEGs related to drug resistance and physiological indices. The asterisk (*) denotes significance at *p* < 0.05; double asterisks (**) indicate *p* < 0.01; triple asterisks (***) indicate *p* < 0.001.

The physiological indicators of *A. alternata* were measured, and it was shown that the CK group had a greater soluble protein content than any other fungicide treatment group, with the MZ treatment group having the lowest levels (Table 8). MDA content, melanin content, and tyrosinase activity all showed a similar trend, with the FLU treatment group having the highest values and all fungicide treatments having greater levels than the CK group. CAT and SOD activity, on the other hand, were highest in the CK group and lowest in the FLU treatment group. The FPKM of fungicide resistance-related DEGs and the FPKM of the physiological indicators mentioned above were analyzed using the Pearson analysis method. The results showed that melanin content, MDA content, and tyrosinase activity were positively correlated with the majority of fungicide resistance-related DEGs. In contrast, soluble protein content, SOD activity, and CAT activity were negatively correlated with the majority of resistance-related DEGs (Figure 10). These findings are consistent with the trends reflected by the physiological indices.

## 4. Discussion

Over the past few decades, *Alternaria* spp. have been the main targets of fungicide application due to their involvement in the development of different foliar diseases in other plants and early blight in potatoes [39,40,41]. Nevertheless, *Alternaria* spp. now exhibit decreased treatment sensitivity and even fungicide resistance as a result of the widespread use of fungicides with various modes of action. Research has demonstrated that the causative agent of potato early blight, *A. solani*, has become resistant to QoI (Quinone outside Inhibitor), DMI, and SDHI (Succinate dehydrogenase inhibitor) fungicides [28,42,43,44]. Similarly, resistance to QoI and SDHI fungicides has also been reported for *A. alternata*, a pathogen that causes leaf diseases in other plants [45,46]. Research on the underlying resistance mechanisms is still scarce even though fungicide resistance in *Alternaria* spp. has been the subject of numerous studies. Transcriptome data from *A. alternata* treated with three fungicides were used in this study to examine potential pathways of fungicide resistance. The findings showed that fungicide stress causes overexpression of fungicide efflux pumps, especially ABC and MFS transporters in the MDR system. Similar transcriptomic analyses in other fungal pathogens, such as *Botrytis cinerea*, *Z. tritici*, and *F. graminearum*, have also identified efflux transporter overexpression as a major resistance mechanism, further supporting the role of these transporters in fungicide resistance [47,48,49]. Specifically, two MFS transporters, *TPO1-1* and ATR1, and one ABC transporter, *ABCB1-1*, were upregulated after FLU treatment; one ABC transporter, *CDR1*, was upregulated after PCZ treatment; one MFS transporter, *TPO1-2*, was upregulated after MZ treatment. Notably, this study did not detect the overexpression of multidrug and toxic compound extrusion families’ (MATEs’) transporters within the MDR system. The transcriptome analysis of *Penicillium italicum*’s reaction to the sterol DMI fungicide PCZ, which found no MATEs in the MDR system, is in line with this finding [50]. In contrast, transcriptomic analysis of *P. digitatum* treated with PCZ revealed the presence of three overexpressed MATE transporters [51]. The absence of MATE overexpression in this study suggests that the potential resistance mechanism of *A. alternata* may not involve MATE transporters.

The ergosterol biosynthesis pathway is another important area of study for fungal fungicide resistance mechanisms. Because *CYP51* is essential for the biosynthesis of ergosterol, a crucial component of the fungal plasma membrane, and because mutations or overexpression of the *CYP51*/*ERG11* protein have been connected to fungicide resistance mechanisms in many fungal species, research on *CYP51* in fungal cytochrome *P450* proteins has been especially prominent [52,53]. In this study, six upregulated DEGs from the *CYP450* gene family were identified outside the ergosterol biosynthesis pathway following treatment with three fungicides. Additionally, overexpression of *CYP51* was observed in the ergosterol biosynthesis pathway in both the FLU and PCZ treatment groups. Transcriptomic studies of azole-resistant strains of *A. fumigatus* and *Fusarium* spp. have similarly reported upregulation of *CYP51*, confirming its critical role in resistance evolution [54,55,56]. Similar findings have been reported in other studies. For example, when resistant isolates of *Alternaria* spp., the potato pathogen, were exposed to the DMI fungicide difenoconazole, *CYP51* induction was identified as a critical determinant of DMI resistance [57]. Additionally, studies using PCZ to treat resistant strains of *P. italicum* revealed the overexpression of ergosterol biosynthesis-related genes *ERG2*, ERG6, and *ERG11* (*CYP51A*), which is consistent with the findings in the present study [51]. In this study, PCZ-treated *A. alternata* strains showed overexpression of *ERG6* and *CYP51*/*ERG11*, but not *ERG2*. These discrepancies may be due to differences in the experimental strains and differences in the strength of their inherent fungicide resistance capabilities.

Other genes and pathways linked to fungal fungicide resistance were also examined in this study. Following treatment of *A. alternata* with MZ, overexpression of glutathione S-transferase (GST) was observed among the stress-resistance and antioxidation-related genes. GSTs have several roles in eukaryotes, including protecting against oxidative damage, detoxifying endogenous and external toxic chemicals, regulating signaling pathways, and taking part in sterol biosynthesis. Thus, GST overexpression is linked to the fungicide resistance of *A. alternata* to MZ [58]. Melanin synthesis in fungi has been shown to play a critical role in stress resistance, particularly in defending against chemical stresses such as high salinity, heavy metals, hydrolytic enzymes, and reactive oxygen species (ROS) [59]. Fungal melanin is characterized by its negative charge, hydrophobicity, high molecular weight, and resistance to chemical degradation, with these properties contributing to its structural concentration in the cell wall [60,61]. The biosynthesis of melanin in fungi primarily involves two pathways—the DOPA and DHN pathways—both of which have been extensively studied in model organisms such as *C. neoformans* and *A. fumigatus* [38]. The DHN pathway, in particular, begins with Malonyl-CoA and leads to the polymerization of 1,8-dihydroxynaphthalene (DHN), producing DHN melanin, with *A. fumigatus* commonly used as a model organism for this process. However, due to the absence of fungal DHN melanin biosynthesis data in the KEGG database, it remains challenging to interpret the pathway’s activity in this study based on the DEGs identified.

In this study, genes related to the DHN melanin biosynthesis pathway in *A. alternata*, including laccase, polyketide synthase, tetrahydroxynaphthalene reductase, and multicopper oxidase, were identified for the first time. These genes’ overexpression was particularly noticeable after FLU treatment, suggesting that *A. alternata*’s DHN melanin production is highly sensitive to FLU. Tyrosinase activity was the main mechanism by which all three fungicide treatments controlled the DOPA melanin production pathway. Tyrosinase upregulation was most strongly caused by FLU treatment, whereas MZ and PCZ treatments had less of an impact. FLU treatment has the greatest upregulated response of the unique tyrosinase responses. These findings suggest that *A. alternata* responds to fungicide treatments by overexpressing melanin-regulating genes, leading to an increase in melanin production. This indicates that melanin synthesis plays an important role as a potential fungal resistance mechanism. Similar findings have been reported in *Aspergillus flavus*, where melanin was shown to protect fungal spores and confer resistance to environmental stress induced by chlorine-based disinfectants. This aligns with the results of the current study, highlighting the critical role of melanin synthesis-regulating genes in fungal stress response and fungicide resistance mechanisms [62].

**Figure 11:**
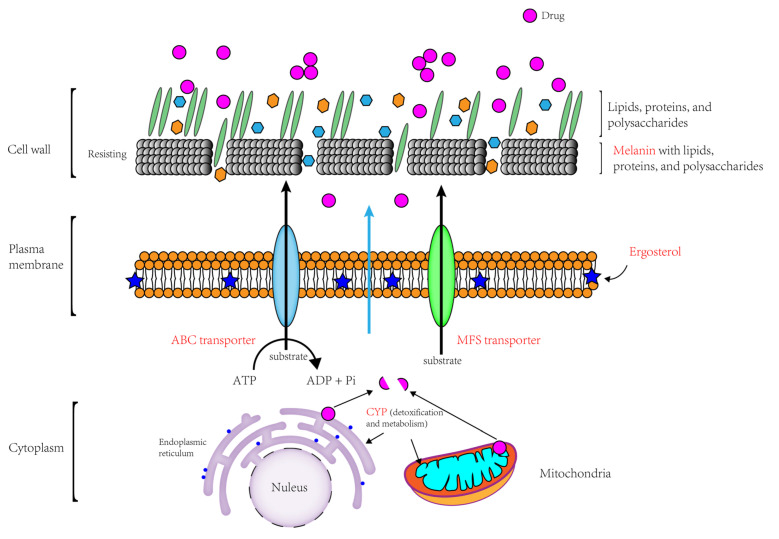
Schematic diagram of the main fungicide resistance mechanisms of *A. alternata*. The purple circle represents medication. Melanin protects cells from chemical degradation by being deposited near the cell wall. The cell wall, strengthened by cross-linked macromolecules, enhances cell rigidity and permeability, thereby mitigating the effects of chemical fungicides by resisting cell swelling. Ergosterol in the cell membrane confers resistance to drugs. ABC transport proteins and MFS transport proteins have an efflux effect on drugs. The CYP450 family on the mitochondrial inner membrane and the endoplasmic reticulum membrane has a metabolic effect on drugs.

This research explored and analyzed the potential fungicide resistance mechanisms of *A. alternata* by investigating transcriptomic data changes induced by fungicide stress, combined with physiological indicators such as tyrosinase and antioxidant enzyme activity. The basic mechanisms of *A. alternata*’s fungicide resistance pathways were clarified for the first time with the proposal of a model (Figure 11). The resistance mechanisms in *A. alternata* are depicted in the model and include the following: (i) Melanin-mediated protection: Melanin deposits near the cell wall, shielding cells from chemical degradation. The cell wall, composed of cross-linked macromolecules, enhances structural strength and rigidity, alters cell permeability, and mitigates cellular swelling, contributing to resistance against chemical fungicides [59,63]. (ii) Ergosterol resistance: Resistance mediated by ergosterol in the fungal cell membrane, which serves as a critical target of fungicides. (iii) Efflux transporters: Overexpression of ABC and MFS transport proteins facilitates the active efflux of fungicides, reducing intracellular fungicide concentrations. (iv) *CYP450*-mediated detoxification: Fungicides are metabolized by the cytochrome P450 family found on the inner membranes of mitochondria and the membranes of the endoplasmic reticulum, which aids in detoxification and resistance [52,64]. This comprehensive model provides novel insights into the multifaceted fungicide resistance mechanisms of *A. alternata* against fungicides.

These findings provide new insights into the complex mechanisms of fungicide resistance in *A. alternata*, which could help improve disease management strategies. The overexpression of ABC and MFS transporters suggests that efflux pump inhibitors may enhance fungicide efficacy. Similarly, the upregulation of *CYP51* highlights the need for fungicide rotation or mixture strategies to minimize resistance selection pressure. Additionally, given the role of melanin biosynthesis in stress response, targeting melanin synthesis pathways may increase fungal susceptibility. Integrating these strategies with biological control and resistant cultivar selection could provide a more sustainable approach to managing fungicide resistance in *A. alternata*.

## 5. Conclusions

Four fungicides that demonstrated notable inhibitory effects were chosen for additional examination after this study examined the susceptibility of the poplar leaf blight pathogen *A. alternata* to twelve chemical fungicides. The EC_50_ values of effective fungicides with different modes of action were determined using a fungicide toxicity regression model. Three of these fungicides—FLU, MZ, and PCZ—showed the best outcomes and were selected for additional study. To identify possible fungicide resistance pathways, the transcriptome data and physiological markers of *A. alternata* under fungicide stress were analyzed. The results showed that while MZ treatment caused the expression of distinct resistance-related genes, *A. alternata* showed comparable resistance mechanisms under FLU and PCZ treatments. The results showed that while MZ treatment caused the expression of distinct resistance-related genes, *A. alternata* showed comparable resistance mechanisms under FLU and PCZ treatments. By mimicking the ergosterol and melanin production pathways, the mechanisms of resistance were better clarified. This study gives a theoretical framework for further research on chemical management and fungicide resistance in *A. alternata*, as well as important insights into the mechanisms underlying this pathogen’s resistance to fungicides.

## Figures and Tables

**Table 1:** Sensitivity rescreening results of *A. alternata* (AaNEFU1) to fungicides based on the mycelial growth inhibition rate.

Fungicide Name	Fungicide Concentration (μg/mL)	2d	3d	4d	5d
Mancozeb	0.1	8.30 ± 0.54% f	3.49 ± 1.50% f	3.13 ± 0.21% f	0.48 ± 0.37% f
	0.5	12.98 ± 1.27% e	5.82 ± 0.56% e	8.70 ± 0.84% e	1.19 ± 0.45% e
	1	15.44 ± 2.19% d	9.41 ± 0.54% d	20.60 ± 0.53% d	21.45 ± 0.27% d
	5	17.89 ± 0.35% c	20.61 ± 0.31% c	34.86 ± 0.21% c	26.38 ± 0.36% c
	10	22.11 ± 0.35% b	33.15 ± 0.94% b	36.74 ± 0.42% b	40.88 ± 0.37% b
	15	72.87 ± 0.73% a	60.30 ± 0.41% a	52.61 ± 0.42% a	46.11 ± 0.27% a
Prochloraz	0.1	21.40 ± 3.86% e	16.31 ± 0.16% f	25.96 ± 0.53% f	23.35 ± 0.36% e
	0.2	36.26 ± 1.58% d	34.77 ± 0.41% e	35.56 ± 0.12% e	36.13 ± 0.10% d
	0.4	40.81 ± 1.42% c	38.80 ± 0.68% d	38.48 ± 0.32% d	36.60 ± 0.27% d
	0.8	48.66 ± 0.20% b	49.10 ± 0.41% c	50.73 ± 0.42% c	49.20 ± 0.36% c
	2	54.97 ± 0.41% a	52.42 ± 0.54% b	54.56 ± 0.32% b	53.24 ± 0.27% b
	4	57.08 ± 0.20% a	55.65 ± 0.27% a	57.83 ± 0.55% a	58.59 ± 0.27% a
Fludioxonil	0.1	29.82 ± 0.70% f	37.10 ± 0.54% f	41.13 ± 0.63% e	34.52 ± 0.37% f
	0.2	32.51 ± 1.07% e	40.50 ± 0.41% e	48.50 ± 0.53% d	50.09 ± 0.18% e
	0.4	41.99 ± 0.54% d	45.61 ± 0.41% d	54.98 ± 0.32% cd	55.08 ± 0.18% d
	0.8	55.67 ± 1.42% c	50.63 ± 0.41% c	57.90 ± 0.12% bc	62.98 ± 0.27% c
	1	60.82 ± 0.54% b	56.36 ± 0.41% b	62.91 ± 0.32% ab	64.77 ± 0.27% b
	2	75.20 ± 0.88% a	72.76 ± 0.41% a	66.53 ± 8.08% a	73.32 ± 0.27% a
Difenoconazole	0.1	17.19 ± 0.70% f	15.50 ± 0.68% f	20.18 ± 0.32% f	19.25 ± 0.36% f
	0.2	24.44 ± 0.88% e	27.78 ± 0.68% e	30.13 ± 0.12% e	32.44 ± 0.47% e
	0.4	27.66 ± 1.23% d	31.00 ± 0.68% d	35.49 ± 0.21% d	35.83 ± 0.36% d
	0.8	41.29 ± 1.23% c	37.28 ± 0.16% c	43.42 ± 0.21% c	42.78 ± 0.18% c
	2	52.28 ± 0.35% b	41.31 ± 0.31% b	45.30 ± 0.21% b	45.75 ± 0.21% b
	4	61.75 ± 1.40% a	53.85 ± 0.16% a	52.61 ± 0.21% a	54.90 ± 0.36% a ^1^

^1^ Significance marking analyzes the difference in the degree of mycelial inhibition under all concentrations of all pesticides with the same mechanism of action during the same period. Letters a–f indicate *p* < 0.05.

**Table 2:** Toxicity regression equation and EC_50_ values for *A. alternata* (AaNEFU1).

Fungicide Name	Regression Equation	r^2^	K	EC_50_ (µg/mL)
Mancozeb	y = 0.0290x + 0.0749	R^2^ = 0.8389	0.0290	14.66
Prochloraz	y = 0.0723x + 0.3381	R^2^ = 0.6979	0.0723	2.24
Fludioxonil	y = 0.1686x + 0.4415	R^2^ = 0.7655	0.1686	0.35
Difenoconazole	y = 0.0692x + 0.2984	R^2^ = 0.7281	0.0692	2.91

**Table 3:** Transcriptome sequencing parameters and alignment rate of *A. alternata* (AaNEFU1).

Samples	Clean Reads	Clean Bases	GC Content	% ≥Q30
CK1	21,861,040	6,535,074,204	54.56%	95.13%
CK2	20,913,041	6,257,953,670	54.38%	95.10%
CK3	19,647,287	5,880,173,540	54.46%	95.29%
Fludioxonil 1	20,434,717	6,113,480,574	54.72%	95.24%
Fludioxonil 2	19,729,983	5,905,210,182	54.98%	95.50%
Fludioxonil 3	19,955,369	5,970,527,032	54.85%	95.08%
Mancozeb 1	21,962,043	6,573,537,506	54.39%	95.02%
Mancozeb 2	23,142,253	6,924,447,538	54.48%	95.09%
Mancozeb 3	20,167,429	6,034,657,224	54.63%	94.99%
Prochloraz 1	20,672,993	6,186,553,534	54.37%	95.20%
Prochloraz 2	20,387,397	6,101,144,570	54.70%	94.87%
Prochloraz 3	22,234,030	6,649,083,082	54.72%	95.19%

**Table 4:** Summary of sequencing data and alignment results to reference genome.

Sample	Total Reads	Mapped Reads	Uniquely Mapped Reads	Multi-Mapped Reads	Positive Strand Mapped Reads	Negative Strand Mapped Reads
CK1	43,722,080	42,296,656 (96.74%)	42,206,36 (96.53%)	90,295 (0.21%)	21,184,372 (48.45%)	21,210,242 (48.51%)
CK2	41,826,082	40,492,095 (96.81%)	40,409,351 (96.61%)	82,744 (0.20%)	20,279,645 (48.49%)	20,303,583 (48.54%)
CK3	39,294,574	38,062,886 (96.87%)	37,988,848 (96.68%)	74,038 (0.19%)	19,059,135 (48.50%)	19,083,660 (48.57%)
Fludioxonil 1	40,869,434	39,448,017 (96.52%)	39,351,670 (96.29%)	96,347 (0.24%)	19,793,641 (48.43%)	19,757,558 (48.34%)
Fludioxonil 2	39,459,966	38,159,320 (96.70%)	38,063,855 (96.46%)	95,465 (0.24%)	19,123,192 (48.46%)	19,138,605 (48.50%)
Fludioxonil 3	39,910,738	38,446,001 (96.33%)	38,354,808 (96.10%)	91,193 (0.23%)	19,263,963 (48.27%)	19,279,011 (48.31%)
Mancozeb 1	43,924,086	42,486,492 (96.73%)	42,379,620 (96.48%)	106,872 (0.24%)	21,342,440 (48.59%)	21,258,963 (48.40%)
Mancozeb 2	46,284,506	44,207,251 (95.51%)	44,106,443 (95.29%)	100,808 (0.22%)	22,165,125 (47.89%)	22,150,397 (47.86%)
Mancozeb 3	40,334,858	38,930,703 (96.52%)	38,848,657 (96.32%)	82,046 (0.20%)	19,500,824 (48.35%)	19,518,747 (48.39%)
Prochloraz 1	41,345,986	40,117,348 (97.03%)	40,016,431 (96.78%)	100,917 (0.24%)	20,157,307 (48.75%)	20,067,135 (48.53%)
Prochloraz 2	40,774,794	39,462,161 (96.78%)	39,375,847 (96.57%)	86,314 (0.21%)	19,770,515 (48.49%)	19,786,084 (48.53%)
Prochloraz 3	44,468,060	42,986,501 (96.67%)	42,884,843 (96.44%)	101,658 (0.23%)	21,541,368 (48.44%)	21,554,549 (48.47%)

**Table 5:** Expression profiles of MDR genes among the DEGs.

Sample Comparison	Gene Expression Trend	ABC Transporters			MFS Transporters	
		Subfamily B	Subfamily C	Subfamily G	DHA1 Family	DHA2 Family
CK vs. FLU	UP	1	0	0	1	1
CK vs. FLU	DOWN	0	1	0	17	1
CK vs. MZ	UP	0	0	0	1	0
CK vs. MZ	DOWN	3	0	0	9	1
CK vs. PCZ	UP	0	0	1	0	0
CK vs. PCZ	DOWN	1	0	0	13	1

**Table 6:** Expression of DEGs in the sterol biosynthesis pathway.

Gene ID	Gene Name	CK vs. FLU log_2_FC	CK vs. MZ log_2_FC	CK vs. PCZ log_2_FC	KEGG Annotation
scaffold29.g33	*ERG1*	−1.61	\	\	squalene monooxygenase
scaffold30.g31	*DHCR24*	1.21	\	\	Delta24-sterol reductase
scaffold70.g28	*ERG4*	−1.21	\	\	Delta24 (24 (1))-sterol reductase
scaffold221.g2	*ERG6*	1.04	\	1.55	sterol 24-C-methyltransferase
scaffold31.g1	*CYP51*	1.02	\	1.05	sterol 14alpha-demethylase
scaffold76.g38	*ERG26-1*	2.65	\	2.18	sterol-4alpha-carboxylate 3-dehydrogenase
scaffold84.g8	*ERG26-2*	\	−1.14	\	sterol-4alpha-carboxylate 3-dehydrogenase
scaffold40.g88	*ERG27*	1.08	\	\	3-keto steroid reductase
scaffold73.g57	*EBP-1*	1.47	\	\	cholestenol Delta-isomerase
*Alternaria_alternata*_newGene_1524	*EBP-2*	−1.24	\	\	cholestenol Delta-isomerase

**Table 7:** Expression of DEGs in the melanin biosynthesis pathway.

Gene ID	Gene Name	CK vs. FLU log_2_ FC	CK vs. MZ log_2_ FC	CK vs. PCZ log_2_ FC	KEGG Annotation
scaffold1.g420	*TYR-1*	1.20	\	\	tyrosinase
scaffold17.g5	*TYR-2*	1.21	\	\	tyrosinase
scaffold3.g176	*TYR-3*	2.29	−1.33	1.11	tyrosinase
scaffold39.g62	*TYR-4*	3.27	\	\	tyrosinase
scaffold44.g51	*TYR-5*	1.23	\	1.02	tyrosinase
scaffold84.g9	*TYR-6*	1.55	−1.36	\	tyrosinase
scaffold99.g40	*TYR-7*	−1.08	\	−1.10	tyrosinase
scaffold44.g82	*TYR-8*	\	3.68	\	tyrosinase
scaffold62.g73	*TYR-9*	\	−1.84	\	tyrosinase
scaffold30.g2	*LAC-1*	−1.09	\	−1.24	laccase
scaffold19.g123	*LAC-2*	1.22	\	\	laccase
scaffold137.g5	*LAC-3*	1.30	−1.29	\	laccase
scaffold20.g78	*LAC-4*	1.70	\	\	laccase
scaffold43.g9	*LAC-5*	\	−2.26	\	laccase
scaffold3.g55	*EASB-1*	1.90	\	\	polyketide synthase
scaffold184.g4	*EASB-2*	−2.08	\	−1.52	polyketide synthase
scaffold106.g31	*ACE1-1*	−1.78	\	\	polyketide synthase
*Alternaria_alternata*_newGene_1437	*ACE1-2*	1.49	\	\	polyketide synthase
scaffold45.g101	*MDPG*	−2.89	−4.35	\	polyketide synthase
scaffold73.g47	*THNR-1*	−1.45	\	\	tetrahydroxynaphthalene reductase
scaffold36.g38	*THNR-2*	−2.00	\	−1.63	tetrahydroxynaphthalene reductase
scaffold78.g52	*OAR1*	\	\	1.15	tetrahydroxynaphthalene reductase
scaffold15.g120	*FET3_5-1*	1.67	\	\	multicopper oxidase
scaffold1.g5	*FET3_5-2*	−1.97	−1.39	−1.17	multicopper oxidase
scaffold36.g36	*FET3_5-3*	−2.51	\	−1.51	multicopper oxidase
scaffold132.g2	*FET3_5-4*	1.33	−1.67	\	multicopper oxidase
scaffold26.g100	*FET3_5-5*	\	−1.56	\	multicopper oxidase

**Table 8:** Physiological index data of *A. alternata* (AaNEFU1).

Sample	CK	FLU	MZ	PCZ
Soluble protein (mg·mL^−1^)	24.35 ± 4.98 c	58.34 ± 3.86 ab	52.30 ± 2.75 b	63.38 ± 1.50 a
Tyrosinase (U·g^−1^)	253.61 ± 18.34 d	598.69 ± 16.19 a	465.21 ± 44.88 c	552.34 ± 11.71 b
Melanin (g·L^−1^)	0.32 ± 0.03 c	0.53 ± 0.01 ab	0.48 ± 0.04 b	0.57 ± 0.06 a
MDA (µmol·g^−1^)	4.67 ± 0.61 c	23.79 ± 3.34 a	14.46 ± 1.45 b	15.88 ± 0.43 b
CAT (U·g^−1^)	27.86 ± 0.48 a	23.62 ± 1.69 b	24.63 ± 1.39 b	25.50 ± 2.08 ab
SOD (U·g^−1^)	155.34 ± 29.73 a	121.70 ± 5.47 a	152.73 ± 15.70 a	150.82 ± 16.08 a ^1^

^1^ Letters a–d indicate *p* < 0.05.

## Data Availability

The original contributions presented in this study are included in the article. Further inquiries can be directed to the corresponding author.

## Abbreviations

The following abbreviations are used in this manuscript:

FST	Fungicide sensitivity tests
MBC	Methyl benzimidazoles
DMIs	demethylation inhibitors
QoIs	Quinone outside Inhibitors
SDHI	Succinate dehydrogenase inhibitor
PPs	Phenylpyrroles
EC_50_	50% effective concentration
MDR	Multidrug resistance
ABC	ATP-binding cassette
MFS	Major facilitator superfamily
FLU	Fludioxonil
MZ	Mancozeb
PCZ	Prochloraz
PDA	Potato dextrose agar
FDR	False discovery rate
DEG	Differentially expressed gene
Log_2_FC	Log2 fold change
FPKM	Fragment Per Kilobase of exon model per Million mapped fragments
GO	Gene Ontology
CC	Cellular Component
MF	Molecular Function
FAD	Flavin adenine dinucleotide
KEGG	Kyoto Encyclopedia of Genes and Genomes
MDA	Malondialdehyde
CAT	Catalase
SOD	Superoxide dismutase
MATE	Multidrug and toxic compound extrusion family
GST	Glutathione S-transferase
ROS	Reactive oxygen species

## Appendix A

**Table A1:** Information on chemical fungicides used.

Fungicide Name and Active Ingredient	Functional Type	Physical Formulation	Manufacturer	Mode of Action Type	Primary Mechanism of Action
50% Difenoconazole	Protective and Therapeutic	Suspension Concentrate	Shandong Libang Agrochemicals Co., Ltd., Qingzhou, China.	DMIs	Inhibits cytochrome P450-dependent 14α-demethylation, blocking ergosterol biosynthesis.
450 g/L Prochloraz	Protective and Eradicant	Emulsifiable Concentrate	Shanghai Hulian Biochemical Co., Ltd., Shanghai, China.	DMIs	Inhibits ergosterol biosynthesis in fungi.
40% Myclobutanil	Protective and Therapeutic	Suspension Concentrate	Jiangsu Yunnong Chemical Co., Ltd., Zhengjiang, China.	DMIs	Inhibits ergosterol biosynthesis in fungi.
50% Carbendazim	Therapeutic	Wettable Powder	Leke Pesticide Co., Ltd., Yingkou, China.	Methyl Benzimidazole Carbamates (MBC)	Prevents cell division by inhibiting tubulin polymerization.
50% Thiophanate-Methyl	Protective	Wettable Powder	Sichuan Runnong Technology Co., Ltd., Jianyang, China.	Methyl Benzimidazole MBC	After plant absorption, decomposes into MBC to disrupt mitotic spindle formation in fungal cells.
75% Chlorothalonil	Protective	Wettable Powder	Chifeng Zhongnongda Biochemistry Technology Co., Ltd., Chifeng, China.	Multi-Site	Disrupts glyceraldehyde-3-phosphate dehydrogenase activity in cells.
80% Mancozeb	Protective	Wettable Powder	Liming Chemical Co., Ltd., Xinyi, China.	Multi-Site	Inhibits pyruvate oxidation in fungi.
80% Zineb	Protective	Wettable Powder	Shandong Xinxing Agrochemicals Co., Ltd., Qingzhou, China.	Multi-Site	Active ingredient oxidizes in water to isothiocyanate, inhibiting -SH group-containing enzymes in fungi.
90% Phosethyl-Al	Therapeutic	Wettable Powder	Liming Chemical Co., Ltd., Xinyi, China.	Multi-Site	Effectively inhibits pyruvate oxidation in fungi.
25 g/L Fludioxonil	Protective	Suspension Concentrate	Syngenta Nantong Crop Protection Co., Ltd., Nantong, China.	PPs	Inhibits glucose-phosphorylation-related transference.
250 g/L Azoxystrobin	Therapeutic	Suspension Concentrate	Syngenta Nantong Crop Protection Co., Ltd., Nantong, China.	QoIs	Binds to the Qo site of the cytochrome bc1 complex, obstructing respiration and leading to fungal pathogen death.
15% Triadimefon	Protective	Wettable Powder	Sichuan Guoguang Agrochemical Co., Ltd., Jianyang, China.	Conazoles	Inhibits ergosterol biosynthesis, thereby impeding appressorium and haustorium development, hyphal growth, and spore formation.

**Table A2:** Preliminary screening results of *A. alternata* (AaNEFU1) to fungicides based on the mycelial growth inhibition rate.

Fungicide Type	Fungicide Name	Fungicide Concentration	Inhibition Rate (%)			
			2d	3d	4d	5d
DMIs	Difenoconazole	0.5	65.16 ± 1.53% i	48.29 ± 0.79% i	58.21 ± 0.12% i	54.90 ± 0.61% k
		5.0	69.68 ± 1.15% g	54.86 ± 0.27% h	63.66 ± 0.12% h	66.67 ± 0.25% i
		10.0	71.45 ± 0.58% f	63.60 ± 0.31% f	71.07 ± 0.21% f	69.87 ± 0.20% h
		50.0	100 ± 0.00% a	100 ± 0.00% a	100.00 ± 0.00% a	100.00 ± 0.00% a
		100.0	100 ± 0.00% a	100 ± 0.00% a	100.00 ± 0.00% a	100.00 ± 0.00% a
	Prochloraz	0.5	67.30 ± 0.22% h	63.69 ± 2.03% f	63.66 ± 0.12% h	65.88 ± 0.17% j
		5.0	87.80 ± 0.21% d	79.27 ± 0.79% d	77.08 ± 0.53% e	74.32 ± 0.34% f
		10.0	89.18 ± 0.95% c	83.46 ± 0.53% c	81.41 ± 1.16% d	79.56 ± 0.00% d
		50.0	100 ± 0.00% a	100 ± 0.00% a	100.00 ± 0.00% a	100.00 ± 0.00% a
		100.0	100 ± 0.00% a	100 ± 0.00% a	100.00 ± 0.00% a	100.00 ± 0.00% a
	Myclobutanil	0.5	53.71 ± 0.33% j	61.94 ± 0.53% g	57.93 ± 0.32% i	54.90 ± 0.17% k
		5.0	69.31 ± 0.12% g	66.76 ± 0.15% e	69.67 ± 0.12% g	71.85 ± 0.26% g
		10.0	75.00 ± 0.00% e	78.84 ± 0.26% d	76.59 ± 0.32% e	74.94 ± 0.10% e
		50.0	91.82 ± 0.50% b	91.51 ± 0.30% b	92.38 ± 0.95% c	86.43 ± 0.10% c
		100.0	100 ± 0.00% a	100 ± 0.00% a	97.55 ± 0.12% b	96.45 ± 0.00% b
MBC	Carbendazim	0.5	7.17 ± 0.38% g	2.45 ± 0.15% f	1.82 ± 0.12% g	7.77 ± 0.17% ef
		5.0	9.94 ± 0.22% f	6.56 ± 0.26% de	8.04 ± 0.12% e	10.19 ± 0.09% d
		10.0	12.83 ± 0.38% e	8.66 ± 0.26% c	11.60 ± 0.12% d	16.95 ± 0.35% c
		50.0	22.39 ± 0.22% b	10.94 ± 0.31% b	14.05 ± 042% c	22.30 ± 0.34% b
		100.0	27.42 ± 0.22% a	15.58 ± 0.15% a	15.79 ± 0.12% b	24.32 ± 0.17% a
	Thiophanate-Methyl	0.5	4.53 ± 0.38% h	5.69 ± 1.24% e	2.10 ± 0.21% g	2.42 ± 0.26% i
		5.0	10.94 ± 0.76% ef	7.44 ± 0.30% d	3.98 ± 0.21% f	4.00 ± 0.09% h
		10.0	17.86 ± 0.58% d	11.37 ± 0.85% b	13.56 ± 0.53% c	5.41 ± 0.61% gh
		50.0	20.25 ± 0.58% c	14.96 ± 0.26% a	18.66 ± 0.00% a	6.36 ± 2.03% fg
		100.0	25.91 ± 2.65% a	15.83 ± 0.55% a	18.73 ± 0.44% a	9.18 ± 0.85% de
Multi-site	Chlorothalonil	0.5	3.40 ± 0.38% n	9.45 ± 0.26% n	9.22 ± 0.21% n	12.44 ± 0.35% k
		5.0	19.37 ± 0.21% k	13.74 ± 1.6% l	14.12 ± 0.53% l	14.75 ± 0.68% j
		10.0	31.70 ± 1.51% g	23.88 ± 0.69% i	20.34 ± 0.55% j	25.06 ± 1.70% g
		50.0	46.29 ± 0.95% d	39.28 ± 0.80% e	43.26 ± 0.12% c	41.27 ± 0.68% c
		100.0	51.57 ± 1.53% c	45.06 ± 0.66% c	46.33 ± 0.63% b	42.63 ± 0.35% b
	Mancozeb	0.5	3.77 ± 0.65% n	17.41 ± 0.66% j	20.96 ± 1.26% j	8.61 ± 0.17% l
		5.0	21.51 ± 0.76% j	43.57 ± 0.26% d	29.07 ± 0.95% f	18.07 ± 0.29% i
		10.0	31.32 ± 0.38% g	49.25 ± 0.15% b	43.82 ± 0.21% c	41.38 ± 1.03% bc
		50.0	100.00 ± 0.00% a	100.00 ± 0.00% a	100.00 ± 0.00% a	100.00 ± 0.00% a
		100.0	100.00 ± 0.00% a	100.00 ± 0.00% a	100.00 ± 0.00% a	100.00 ± 0.00% a
	Zineb	0.5	19.37 ± 0.58% k	17.76 ± 0.15% j	22.08 ± 0.12% i	22.64 ± 0.17% h
		5.0	21.26 ± 0.22% j	27.56 ± 0.26% h	24.95 ± 0.21% h	25.00 ± 0.17% g
		10.0	25.66 ± 0.38% i	30.54 ± 0.15% g	26.97 ± 0.12% g	26.97 ± 0.10% f
		50.0	35.85 ± 0.38% f	31.76 ± 1.46% g	30.40 ± 0.21% e	36.38 ± 0.10% e
		100.0	59.75 ± 0.22% b	43.83 ± 0.26% cd	39.41 ± 0.42% d	37.84 ± 0.17% d
	Phosethy-Al	0.5	11.83 ± 0.22% m	9.19 ± 0.27% n	2.52 ± 0.42% p	0.56 ± 0.25% o
		5.0	16.60 ± 0.38% l	11.29 ± 0.27% m	7.97 ± 0.42% o	1.52 ± 1.27% o
		10.0	19.75 ± 0.43% k	15.40 ± 0.40% k	11.53 ± 0.21% m	4.00 ± 0.09% n
		50.0	29.06 ± 0.38% h	17.59 ± 0.27% j	14.47 ± 0.21% l	6.31 ± 0.10% m
		100.0	37.49 ± 0.44% e	36.22 ± 0.53% f	19.22 ± 0.32% k	8.95 ± 0.68% l
PPs	Azoxystrobin	0.5	66.54 ± 0.43% b	59.84 ± 0.26% d	56.81 ± 0.21% c	48.99 ± 0.17% c
		5.0	100.00 ± 0.00% a	94.84 ± 0.40% c	94.69 ± 0.12% b	92.17 ± 0.10% b
		10.0	100.00 ± 0.00% a	96.98 ± 0.15% b	100.00 ± 0.00% a	100.00 ± 0.00% a
		50.0	100.00 ± 0.00% a	100.00 ± 0.00% a	100.00 ± 0.00% a	100.00 ± 0.00% a
		100.0	100.00 ± 0.00% a	100.00 ± 0.00% a	100.00 ± 0.00% a	100.00 ± 0.00% a
QoIs	Azoxystrobin	0.5	35.47 ± 1.13% e	27.30 ± 0.53% e	27.81 ± 2.45% e	15.48 ± 1.61% e
		5.0	39.50 ± 1.53% d	34.73 ± 1.45% d	32.77 ± 0.12% d	17.34 ± 0.35% d
		10.0	42.26 ± 0.38% c	38.06 ± 0.00% c	34.94 ± 0.12% c	23.14 ± 1.50% c
		50.0	48.30 ± 0.65% b	40.07 ± 0.15% b	40.18 ± 0.12% b	25.40 ± 0.10% b
		100.0	52.08 ± 0.38% a	41.30 ± 0.15% a	42.00 ± 0.12% a	39.42 ± 0.10% a
Conazoles	Triadimefon	0.5	7.30 ± 0.22% e	0.88 ± 0.15% e	2.73 ± 0.21% e	11.54 ± 0.52% e
		5.0	29.43 ± 0.38% d	28.70 ± 0.30% d	26.21 ± 0.21% d	28.78 ± 0.10% d
		10.0	37.36 ± 0.38% c	31.41 ± 0.40% c	32.42 ± 0.12% c	35.58 ± 0.10% c
		50.0	50.69 ± 1.16% b	42.34 ± 0.40% b	39.13 ± 0.74% b	41.55 ± 0.17% b
		100.0	56.73 ± 2.79% a	51.53 ± 0.16% a	43.75 ± 0.24% a	45.67 ± 0.10% a ^1^

^1^ Significance marking analyzes the difference in the degree of mycelial inhibition under all concentrations of all pesticides with the same mechanism of action during the same period. Letters a–m indicate *p* < 0.05.
